# Antidiabetic DPP-4 Inhibitors Reprogram Tumor Microenvironment That Facilitates Murine Breast Cancer Metastasis Through Interaction With Cancer Cells *via* a ROS–NF-кB–NLRP3 Axis

**DOI:** 10.3389/fonc.2021.728047

**Published:** 2021-09-24

**Authors:** Rui Li, Xin Zeng, Meihua Yang, Jinmei Feng, Xiaohui Xu, Liming Bao, Tingbo Ye, Xin Wang, Bingqian Xue, Yi Huang

**Affiliations:** ^1^ National Clinical Research Center for Child Health and Disorders, Ministry of Education Key Laboratory of Child Development and Disorders, Chongqing Key Laboratory of Child Infection and Immunity, Children’s Hospital of Chongqing Medical University, Chongqing, China; ^2^ Departments of Neurology, Washington University School of Medicine and Barnes-Jewish Hospital, Saint Louis, MO, United States; ^3^ Department of Pathology, School of Medicine, University of Colorado Anschutz Medical Campus, Aurora, CO, United States; ^4^ Department of Laboratory Medicine, The Third People’s Hospital of Chengdu, Chengdu, China; ^5^ Department of Laboratory Medicine, The People’s Hospital of Guangxi Zhuang Autonomous Region, Nanning, China; ^6^ Department of Laboratory Medicine, Chengdu Women’s and Children’s Central Hospital, Chengdu, China

**Keywords:** DPP-4 inhibitors, breast cancer, NF-кB, NLRP3 inflammasome, metastasis, tumor microenvironment

## Abstract

Improvement of understanding of the safety profile and biological significance of antidiabetic agents in breast cancer (BC) progression may shed new light on minimizing the unexpected side effect of antidiabetic reagents in diabetic patients with BC. Our recent finding showed that Saxagliptin (Sax) and Sitagliptin (Sit), two common antidiabetic dipeptidyl peptidase-4 inhibitors (DPP-4i) compounds, promoted murine BC 4T1 metastasis *via* a ROS–NRF2–HO-1 axis in nonobese diabetic–severe combined immunodeficiency (NOD-SCID) mice. However, the potential role of DPP-4i in BC progression under immune-competent status remains largely unknown. Herein, we extended our investigation and revealed that Sax and Sit also accelerated murine BC 4T1 metastasis in orthotopic, syngeneic, and immune-competent BALB/c mice. Mechanically, we found that DPP-4i not only activated ROS–NRF2–HO-1 axis but also triggered reactive oxygen species (ROS)-dependent nuclear factor kappa B (NF-κB) activation and its downstream metastasis-associated gene levels *in vitro* and *in vivo*, while NF-кB inhibition significantly abrogated DPP-4i-driven BC metastasis *in vitro*. Meanwhile, inhibition of NRF2–HO-1 activation attenuated DPP-4i-driven NF-кB activation, while NRF2 activator ALA enhanced NF-кB activation, indicating an essential role of ROS–NRF2–HO-1 axis in DPP-4i-driven NF-кB activation. Furthermore, we also found that DPP-4i increased tumor-infiltrating CD45, MPO, F4/80, CD4, and Foxp3-positive cells and myeloid-derived suppressor cells (MDSCs), and decreased CD8-positive lymphocytes in metastatic sites, but did not significantly alter cell viability, apoptosis, differentiation, and suppressive activation of 4T1-induced splenic MDSCs. Moreover, we revealed that DPP-4i triggered ROS-NF-κB-dependent NLRP3 inflammasome activation in BC cells, leading to increase in inflammation cytokines such as interleukin (IL)-6, tumor necrosis factor alpha (TNF-α), vascular endothelial growth factor (VEGF), intercellular cell adhesion molecule 1 (ICAM-1), vascular cell adhesion molecule 1 (VCAM-1), IL-1β and IL-33, and MDSCs inductors granulocyte-macrophage colony-stimulating factor (GM-CSF), G-CSF, and M-CSF, which play a crucial role in the remodeling of tumor immune-suppressive microenvironment. Thus, our findings suggest that antidiabetic DPP-4i reprograms tumor microenvironment that facilitates murine BC metastasis by interaction with BC cells *via* a ROS–NRF2–HO-1–NF-κB–NLRP3 axis. This finding not only provides a mechanistic insight into the oncogenic ROS–NRF2–HO-1 in DPP-4i-driven BC progression but also offers novel insights relevant for the improvement of tumor microenvironment to alleviate DPP-4i-induced BC metastasis.

## Introduction

Accumulating evidence indicates that diabetes enhances the incidence of human cancers including breast cancer (BC) (1). Long-term exposure of antidiabetic reagents may have unexpected effects on diabetic patients with BC. Thus, better understanding of the safety profile and biological significance of antidiabetic agents in BC progression is essential to minimize the side effect of antidiabetic agents in BC bearing-diabetic patients (2). Dipeptidyl peptidase-4 inhibitors (DPP-4i), as one of common antidiabetic reagents, are currently recommended for the first-line hypoglycemic treatment of type 2 diabetes mellitus (T2DM). Emerging evidence recently reported an unpredictable adverse effect of DPP-4i in cancer progression (3–6). Our latest finding also revealed that Saxagliptin (Sax) and Sitagliptin (Sit), two common antidiabetic DPP-4i reagents, facilitated murine 4T1 BC cells metastasis in immune-deficient nonobese diabetic–severe combined immunodeficiency (NOD-SCID) mice (7). However, the risky effect of DPP-4i on BC progression under immune-competent status remains largely unknown.

The concept of cancer immunoediting offers a novel insight into the crosstalk between tumor cells and immune system during the cancer progression (8, 9). Tumor microenvironment, including tumor immune microenvironment, has been recognized as a complex milieu where tumor cells interact with immune cells *via* numerous biochemical and physical signals that are crucial for cancer progression (10, 11). Tumor cells, as a major orchestrator of tumor microenvironment, have been shown to reprogram tumor microenvironment by producing cytokines and even inducing or recruiting the immunosuppressive cells such as regulatory T (Treg) cells or myeloid-derived suppressor cells (MDSCs) during cancer progression (7–13). Although recent data suggest a potential role of DPP-4 inhibition in CXCL10-mediated lymphocyte trafficking in melanoma B16F10-bearing mice (12), very little information is available for the potential effect of DPP-4i on tumor immune microenvironment, especially on tumor-infiltrating immune-suppressive cells in BC progression.

In the present study, we utilized the orthotopic and syngeneic murine 4T1 BC metastasis model in immune-competent BALB/C mice, a well-known mice model to effectively mobilize MDSCs, to characterize the effect of Sax and Sit on BC metastasis under immune-competent conditions. Then, we further investigated whether and how DPP-4i can reprogram tumor microenvironment during the BC metastasis.

## Materials and Methods

### Cell Culture and Reagents

Murine BC 4T1 cell line was maintained and cultured as our previous reports (7, 13). Sax (0, 0.2, and 0.4 μM) and Sit (0, 0.6, and 1.2 μM) (MCE, Houston, USA), NRF2 specific inhibitor ML-385 (0, 5, and 10 µM) (MCE, Houston, USA), Heme oxygenase 1 (HO-1) specific inhibitor HO-1-IN-1 hydrochloride (0, 5, and 10 µM) (MCE, Houston, USA), reactive oxygen species (ROS) scavenger N-acetylcysteine (NAC) (0, 2.5, and 5 mM) (AbMole, Houston, USA), and NRF2 activator alpha-lipoic acid (ALA) (0, 40, and 60 μM) (Dandong Yichuang Co, China) were used as described previously (7). pNF-кB-TA-luc reporter plasmids were obtained from Beyotime, Jiangsu, China, as described previously (14). Granulocyte-macrophage colony-stimulating factor (GM-CSF) was purchased from PeproTech, Cranbury, USA. NLRP3 inhibitor MCC950 (0, 5, and 10 µM) (MCE, Houston, USA), caspase-1 inhibitor VX-765 (0, 10, and 20 µM) (AbMole, Houston, USA), and nuclear factor kappa B (NF-κB) specific inhibitor BAY 11-7082 (0, 2, and 4 µM) (Selleck, Houston, USA) were used in this study. For pharmacological intervention assays, 4T1 cells were pretreated with Sax (0.4 μM) or Sit (1.2 μM) for 16 h and then cotreated with indicated inhibitors or activators for additional 4–6 h unless otherwise specified. All chemical reagents were purchased from Sigma-Aldrich (St. Louis, MO, USA) unless otherwise indicated.

### Cell Migration and Cell Invasion Assays

Cell migration and cell invasion assays were performed in 24-well non-coated or Matrigel-coated Transwell chambers [8-µm pore size, Corning, NY, USA as described previously (6, 7, 14, 15)]. Briefly, 4.5 × 10^4^ cells (for cell migration) or 1 × 10^5^ cells (for cell invasion) were seeded in the upper chamber with 200 μl of serum-free medium, and 800 μl medium supplemented with 10% fetal bovine serum (FBS) was used as a chemoattractant in the bottom chamber, and then treated with Sax (0, 0.2, and 0.4 μM) or Sit (0, 0.6, and 1.2 μM) for 24 h. Migration or invasion cells were then fixed and stained with Crystal Violet Staining Solution (Beyotime, Haimen, China). For pharmacological intervention in this assay, 4T1 cells were cotreated with Sax (0.4 μM) or Sit (1.2 μM) and indicated reagents for 24 h. The images of the migrated or invaded cells were captured, and cell number was counted in 5–10 random fields for each group and summarized as mean ± standard deviation (SD) for statistical analysis.

### Spontaneous Orthotopic and Syngeneic 4T1 BC Metastasis Mouse Models

NOD-SCID mice (6–8 weeks, female, SPF degree, 22 ± 3 g) were purchased from Beijing HFK Bioscience Co. (Beijing, China). Wild-type BALB/c mice (6–8 weeks, female, SPF degree, 22 ± 3 g) were purchased from Animal Center of Chongqing medical University (Chongqing, China). All mice were housed and maintained under specific pathogen-free (SPF) conditions as described previously (7, 14, 15). Spontaneous orthotopic BC metastasis models in NOD-SCID or BALB/c mice were established as previously described (7, 13). In brief, 4T1 cells (1 × 10^5^) in 100 µl phosphate-buffered saline (PBS) were subcutaneously injected into in the left mammary fat pad of NOD-SCID or BALB/c mice. After 3–5 days, 4T1-bearing NOD-SCID mice were randomly divided into two groups to receive 0.9% NaCl or Sax (15 mg/kg) *via* oral gavage daily (n = 3–5 mice/group) for 2 weeks as our described previously (7), while 4T1-bearing BALB/c mice were randomly divided into three groups to receive 0.9% NaCl, Sax (15 mg/kg), or Sit (120 mg/kg) *via* oral gavage daily (n = 3–5 mice/group) for 2 weeks. For ALA intervention *in vivo*, 4T1-bearing NOD-SCID mice were randomly divided into two groups to receive intraperitoneal (i.p.) administration of 0.9% NaCl or ALA (80 mg/kg in 0.9% NaCl) three times per week (n = 3–5 mice/group) for 2 weeks as described previously (7). At the end of experiments, mice were sacrificed, and peripheral blood, spleen, and liver and lung tissues were harvested for further analysis. All procedures were approved by the Institutional Animal Care and Use Committee of Children’s Hospital of of Chongqing Medical University.

### Reactive Oxygen Species Detection

Intracellular ROS and mitochondrial ROS (mROS) were measured by flow cytometry as described previously (7, 15). Briefly, Sax- or Sit-treated cells were stained with dihydroethidium (DHE,10 μM) (Sigma-Aldrich) for 30 min at 37°C and were resuspended in ice-cold PBS for intracellular ROS analysis by flow cytometry. For mROS detection, Sax- or Sit-treated cells were stained with MitoSoX Red probe (5.0 μM, Thermo Fisher Scientific) for 20 min at 37°C. After washing with PBS, mROS were analyzed by flow cytometry.

### RNA Isolation and Quantitative Real-Time PCR

RNA isolation and quantitative real-time PCR (qRT-PCR) were performed as described previously (7, 14, 15). Briefly, total RNA was isolated from cells using Tripure Isolation Reagent (Roche, Mannheim, Germany). One microgram of total RNA was reverse transcribed into complementary DNA (cDNA) using the PrimeScript™ RT reagent Kit with gDNA Eraser (Takara, Japan), and qRT-PCR was performed with QuantiNova SYBR Green PCR Kit (Qiagen, Germany) on CFX Connect™ Real-Time System (BIO-RAD) according to the manufacturer’s instructions. The relative gene expressions were normalized to the housekeeping β-actin gene and calculated using the 2^−ΔΔCt^ method. The details of the primers are listed in **Supplementary Table S1**.

### Western Blotting

4T1 cells were treated with indicated reagents and then subject to Western blotting analysis as described previously (7, 13–18). In brief, protein lysates extracted using radioimmunoprecipitation assay (RIPA) buffer (Beyotime, Haimen, China) were resolved by sodium dodecyl sulfate–polyacrylamide gel electrophoresis (SDS-PAGE) by blotting onto polyvinylidene fluoride (PVDF) membranes, blocked in QuickBlock™ Blocking Buffer (Beyotime, Haimen, China), and followed by primary antibody incubation at 4°C overnight. After washing with TBST buffer, blots were incubated with horseradish peroxidase-conjugated secondary antibodies for 1.0 h, washed with TBST three times, and detected with the enhanced chemiluminescence (ECL) system. All antibodies used in this study are listed in **Supplementary Table S2**.

### Luciferase Reporter Gene Assays

Luciferase reporter gene assay was performed as described previously (7, 14, 15). Briefly, cells (1 × 10^4^ cells per well) were seeded in 96-well plates and then transfected with the pNF-кB-TA-luc vectors (90 ng) and pRL-TK *Renilla* plasmids (10 ng) (Promega, Madison, USA) using X-tremeGENE HP DNA Transfection Reagent (Roche, Germany). After transfection 16–18 h, indicated reagents were added for additional 24 h incubation, and the Firefly and Renilla luciferase activities were quantified using the Dual-Glo^®^ Luciferase Assay System (Promega, Madison, USA). The relative luciferase (Luc) activity was present as the fold change of in Firefly luciferase activity after normalization to the Renilla luciferase activity.

### MDSCs Purification, Cell Culture, and Detection of Cell Differentiation

CD11b^+^Gr-1^+^ MDSCs were isolated from splenic cells of 4T1-bearing BALB/c mice using myeloid-derived suppressor cell isolation kit (Miltenyi, Germany) according to the manufacturer’s instructions. Purified MDSCs were co-cultured with Sax (0, 0.2, and 0.4 μM) or Sit (0, 0.6, and 1.2 μM) in the presence of GM-CSF (10 ng/ml) for 72–96 h. MDSCs differentiation *in vitro* was evaluated by analyzing monocytic (Mo)-MDSCs (Ly6C^hi^Ly6G^−^) and granulocytic (G)-MDSCs (Ly6C^lo^Ly6G^+^) using flow cytometry. For *in vivo* analysis, cell percentages of MDSCs in peripheral blood mononuclear cells (PBMCs) and splenic cells were evaluated by gating on CD11b^+^ and Gr-1^+^ population using flow cytometry. MDSCs differentiation *in vivo* was evaluated by analyzing Mo-MDSCs and G-MDSCs in PBMCs or splenic cells after gating on CD11b^+^ population using flow cytometry. Fluorescence-conjugated antibodies for flow cytometry are listed in **Supplementary Table S3**.

### Cell Viability and Cell Apoptosis Assays

Cell viability and cell apoptosis were performed as described previously (14). Briefly, purified MDSCs (1 × 10^3^/well, 96-well plate) were co-cultured with Sax (0, 0.2, and 0.4 μM) or Sit (0, 0.6, and 1.2 μM) in the presence of GM-CSF (10 ng/ml) for 24–96 h and then subject to cell viability or cell apoptosis analysis. Cell viability (co-cultured for 24–96 h) was measured using the enhanced Cell Counting Kit-8 (CCK-8) (Beyotime, Jiangsu, China), and cell apoptosis (co-cultured for 72-96 h) was performed by flow cytometry using Annexin V-PE/7-AAD Apoptosis Kit (KeyGEN Biotech, Nanjing, China) according to the manufacturer’s instructions.

### Flow Cytometry

All flow cytometry analysis were performed on a FACS Calibur flow cytometer (BD Bioscience) and data analyzed with FlowJo software (Tree Star, Ashland, OR) as described previously (6, 13–15).

### H&E Staining and Immunohistochemistry

H&E and immunohistochemistry (IHC) staining were performed as described previously (6, 7, 13–18). Briefly, liver or lung metastatic tissues were fixed with 10% buffered formalin and embedded in paraffin, and tissue sections (4 μm) were used for H&E staining. IHC staining was performed using Elivision plus Polyer HRP IHC Kit (Maixin, Fujian, China) and DAB kit (ZSGB-Bio, Beijing, China) according to the manufacturer’s instructions. All antibodies are listed in **Supplementary Table S2**.

### Immunofluorescence

Indirect or direct immunofluorescence (IF) staining was performed in paraffin-embedded liver or lung metastatic tissue sections (4 μm) as described previously (6, 14, 15). In brief, tissue sections were blocked with QuickBlock™ Blocking Buffer (Beyotime, Haimen, China) for 15 min at room temperature. Then, indirect IF staining was performed to detect CD45, CD4, CD8, MPO, and CD11b by incubating with primary antibodies at 4°C overnight, followed by incubation for 1–2 h at room temperature with AF555- or AF647-conjugated secondary antibody (Bioss, Beijing, China). Direct IF double staining was performed to detect CD11b/F4/80 and CD11b/Gr-1 by incubating with fluorescence-conjugated primary antibodies at 4°C overnight. Nuclei were counterstained with 4′,6-diamidino-2-phenylindole (DAPI). Images were captured using a Nikon AIR Confocal Laser Microscope (Nikon, Minato, Japan), and data were measured by a NIS elements AR analysis software version 5.21. All antibodies are listed in **Supplementary Table S3**.

### Statistics

Statistical analysis was carried out with the GraphPad Prism 7.0 (GraphPad Software) as previously described (7, 13–19). All data were expressed as means ± SD. The significance of difference between groups was determined by unpaired two-tailed Student’s t-test or one-way analysis of variance (ANOVA). The value of p < 0.05 was considered statistically significant.

## Results

### DPP-4i (Sax and Sit) Facilitates 4T1 BC Cells Metastasis in Immune-Competent BALB/c Mice

**Figure 1:**
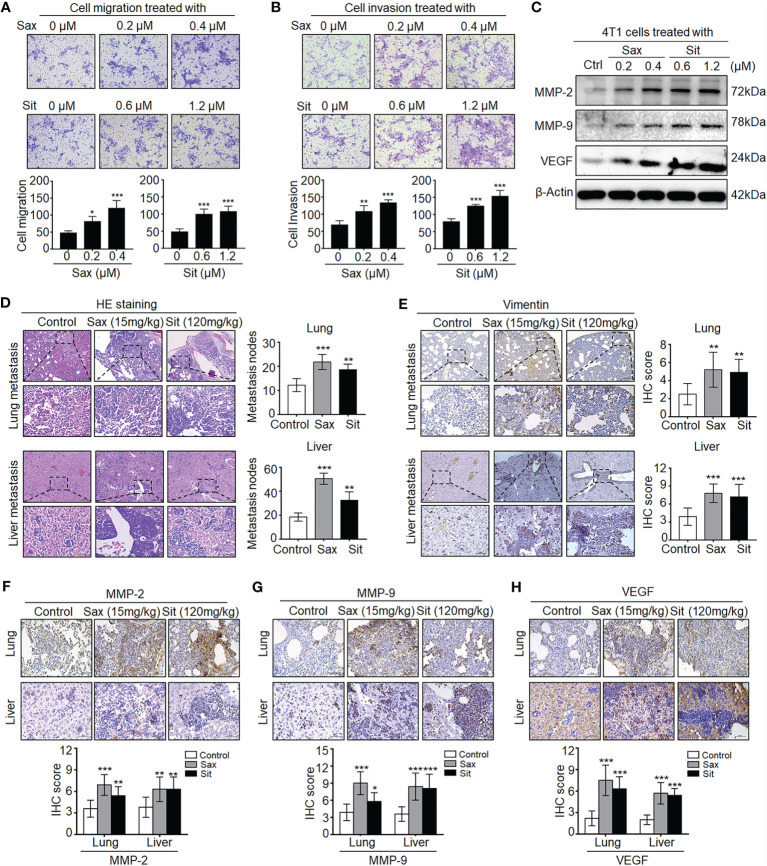
DPP-4i (Sax and Sit) promotes murine breast cancer‬ metastasis in immune-competent BALB/c mice. **(A, B)** DPP-4i (Sax and Sit) facilitates BC cells metastasis *in vitro*. The metastatic ability of 4T1 cells was evaluated by **(A)** cell migration and **(B)** cell invasion assays upon treatment of Sax (0, 0.2, and 0.4 μM) or Sit (0, 0.6, and 1.2 μM) for 24 h. Migration or invasion cells were counted in 5–10 random fields (200× magnification). **(C)** DPP-4i increases metastasis-associated gene levels. Metastasis-associated MMP-2, MMP-9 and VEGF levels were detected by Western blotting in DPP-4i-treated 4T1 cells. β-Actin gene was as a loading control. **(D, E)** DPP-4i accelerates BC lung and liver metastasis *in vivo*. 4T1 cells (1.0 × 10^5^) in 100 μl PBS buffer were injected into in the left mammary fat pad of female BALB/c mice. Postinjection 3–5 days, mice were randomly allocated to three groups (n = 3–5 mice/group) and then treated with 0.9% NaCl (control), Sax (15 mg/kg), or Sit (120 mg/kg) *via* oral gavage daily. After 2 weeks, lung and liver tissues were collected, and metastatic nodes were analyzed by **(D)** H&E staining and **(E)** IHC staining for vimentin. **(F–H)** DPP-4i enhances metastasis-associated gene expression *in vivo*. MMP-9, MMP-2, and VEGF were detected by IHC staining in lung and liver metastatic tissues. Data are presented as mean ± SD of three independent experiments. Representative images are shown. Scale bar: 200 μm (low magnification); 50 μm (high magnification). *p < 0.05, **p < 0.01, and ***p < 0.001 between the indicated groups determined by the one-way analysis of variance (ANOVA).

To understand the potential role of DPP-4i in BC metastasis, we investigated the effect of Sax and Sit, two DPP-4i compounds, on BC metastasis *in vitro* and *in vivo*. We found that Sax and Sit markedly promoted cell migration and cell invasion of BC cells (**Figures 1A, B** **)**, consistent with our previous finding (7). Meanwhile, metastasis-associated proteins MMP-2, MMP-9 and vascular endothelial growth factor (VEGF) were also significantly enhanced after DPP-4i treatment (**Figure 1C**), consistent with their messenger RNA (mRNA) levels upon DPP-4i treatment (7). These results indicate that DPP-4i promotes BC metastasis *in vitro*.

Given an oncogenic role of Sax in immune-deficient NOD-SCID mice (7), we sought to know whether DPP-4i could accelerate 4T1 BC metastasis in immune-competent BALB/c mice. As shown in **Figures 1D, E**, we observed that treatment of Sax and Sit significantly enhanced lung and liver metastasis of BC cells *in vivo* as shown in HE and IHC staining for micro-metastasis marker vimentin. Moreover, metastasis-associated MMP-2, MMP-9, and VEGF expressions were further detected by IHC staining in lung and liver micro-metastasis nodes (**Figures 1F–H**). These results suggest that DPP-4i facilitates spontaneous metastasis of BC cells in immune-competent BALB/c mice.

### DPP-4i Induces ROS-Dependent NF-кB Activation in BC Cells

**Figure 2:**
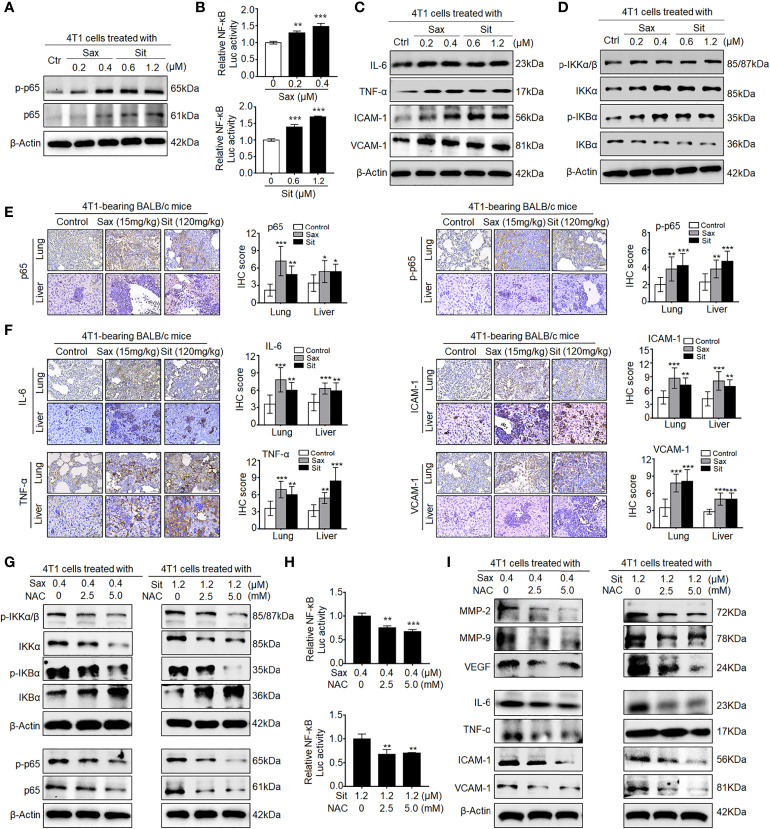
DPP-4i triggers aberrant NF-кB activation *via* a ROS-dependent manner. **(A–D)** DPP-4i induces aberrant NF-кB activation of 4T1 cells *in vitro*. 4T1 cells were treated with Sax (0, 0.2, and 0.4 μM) or Sit (0, 0.6, and 1.2 μM) for 24 h. **(A)** Total p65, p-p65, **(C)** NF-кB-responsive proteins, and **(D)** p-IKKα/β, IKKα, p-IKBα, and IKBα levels were detected by Western blotting, and **(B)** NF-кB transcriptional activation was analyzed by luciferase reporter assay. **(E, F)** DPP-4i enhances aberrant NF-кB activation of 4T1 cells *in vivo*. 4T1-bearing BALB/c mice were treated with or without DPP-4i. **(E)** Total p65, p-p65, and **(F)** NF-кB-responsive proteins were detected in lung and liver metastatic tissues by IHC staining. **(G–I)** ROS scavenger abrogates DPP-4i-driven NF-кB activation *in vitro*. 4T1 cells were co-treated with Sax (0.4 μM) or Sit (1.2 μM) and NAC (0, 2.5, and 5 mM), respectively. **(G)** Total p65, p-p65, p-IKKα/β, IKKα, p-IKBα, and IKBα and **(I)** NF-кB-responsive proteins were detected by Western blotting. **(H)** NF-кB transcriptional activation was analyzed by luciferase reporter assay. β-Actin was as a loading control. Data are presented as mean ± SD of three independent experiments. Representative images are shown. Scale bars: 50 μm. *p < 0.05, **p < 0.01 and ***p < 0.001 between the indicated groups determined the one-way analysis of variance (ANOVA).

Since DPP-4i-induced MMP-2, MMP-9, and VEGF were identified as NF-кB-responsive targets (7, 14), we further sought to know whether NF-кB activation is involved in DPP-4i-induced BC metastasis. *In vitro*, we found that DPP-4i treatment significantly increased NF-кB-p65 (p65) and phosphorated p65-ser536 (p-p65) expression (**Figure 2A**) and NF-кB transcriptional activation (**Figure 2B**). Meanwhile, NF-кB-responsive IL-6, tumor necrosis factor alpha (TNF-α), intercellular cell adhesion molecule 1 (ICAM-1), and vascular cell adhesion molecule 1 (VCAM-1) cytokines were also significantly upregulated upon DPP-4i treatment (**Figure 2C**). Furthermore, we found that DPP-4i significantly altered p-IKKα/β, IKKα, p-IKBα, and IKBα levels (**Figure 2D**), indicating that DPP-4i-induced NF-кB activation is IKK/IKBα dependent. In complementary 4T1-bearing BALB/c mice, we further observed increased levels of p65, p-p65, and NF-кB-responsive IL-6, TNF-α, ICAM-1, and VCAM-1 in lung and liver metastasis tissues after DPP-4i treatment (**Figures 2E, F** **)**. Moreover, in 4T1-bearing NOD-SCID mice, we also observed that Sax treatment also enhanced IL-6, TNF-α, ICAM-1, and VCAM-1 levels in lung and liver metastasis tissues (**Supplementary Figure S1**). Thus, these data suggest that DPP-4i triggers aberrant NF-кB activation *in vitro* and *in vivo*.

Given the aberrant ROS in DPP-4i-treated BC cells (7), we further investigated whether ROS inhibition could reverse DPP-4i-driven NF-кB activation in BC cells. As shown in **Figure 2G**, we found that ROS scavenger NAC significantly reversed DPP-4i-induced p-IKKα/β, IKKα, p-IKBα, and IKBα expressions and p65 and p-p65 levels. Furthermore, NF-кB transcriptional activation and NF-кB-responsive targets levels were significantly attenuated after NAC treatment in DPP-4i-treated BC cells (**Figures 2H, I** **)**. Collectively, these data indicate that DPP-4i induces aberrant NF-кB activation in BC cells *via* a ROS-dependent manner.

### Inhibition of ROS–NF-кB Activation Abrogates DPP-4i-Driven BC Metastasis

**Figure 3:**
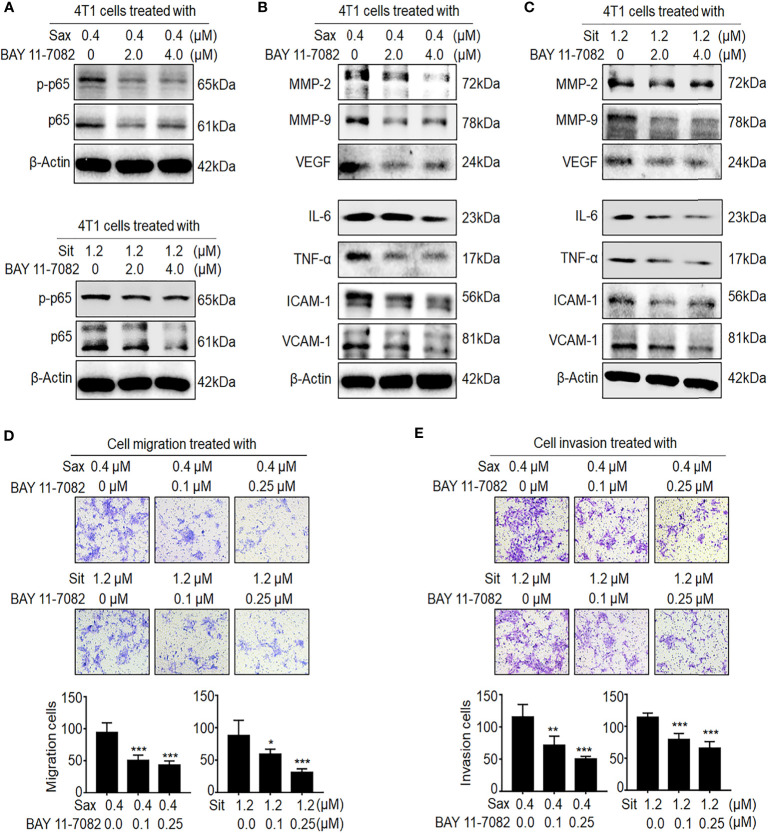
Blockage of NF-кB activation reverses DPP-4i-driven metastasis *in vitro.* **(A–C)** NF-кB inhibitor reverses DPP-4i-driven NF-кB activation. 4T1 cells were co-treated with Sax (0.4 μM) or Sit (1.2 μM) and NF-кB inhibitor BAY 11-7082 (0, 2, and 4 μM), respectively. **(A)** Total p65 and p-p65 expressions and **(B, C)** NF-кB-responsive genes were detected by Western blotting. **(D, E)** NF-кB blockage attenuates DPP-4i-driven cell migration and invasion *in vitro*. 4T1 cells were subject to **(D)** cell migration and **(E)** cell invasion upon co-treatment with Sax (0.4 μM) or Sit (1.2 μM) and BAY 11-7082 (0, 0.1, and 0.25 μM) for 24 h. Migration or invasion cells were counted in 5–10 random fields. Data are presented as mean ± SD of three independent experiments. Representative images are shown. *p < 0.05, **p < 0.01, and ***p < 0.001 between the indicated groups determined by unpaired Student’s t-test or the one-way analysis of variance (ANOVA).

Next, we investigated whether ROS–NF-кB activation is critical to DPP-4i-induced BC metastasis. Using ROS scavenger NAC, we found that ROS inhibition significantly abrogated DPP-4i-driven BC cell migration and invasion with a dose-dependent manner (**Supplementary Figure S2**), consistent with our previous finding (7), suggesting an oncogenic role of ROS in DPP-4i-driven BC metastasis. To further define the role of NF-кB activation in DPP-4i-driven BC metastasis, we used BAY 11-7082, a specific NF-кB inhibitor to explore whether pharmaceutical NF-кB inhibition could reverse DPP-4i-driven BC metastasis. As shown in **Figure 3A**, we found that NF-кB inhibition significantly attenuated p65 and p-p65 levels and NF-кB-responsive and metastasis-associated proteins in DPP-4i-treated BC cells (**Figures 3B, C** **)**. Notably, DPP-4i-driven cell migration and invasion were also significantly abrogated by NF-кB inhibition with a dose-dependent manner (**Figures 3D, E** **)**, indicating an essential role of NF-кB activation in DPP-4i-driven BC metastases *in vitro*. Therefore, these results suggest that DPP-4i drives BC metastasis *via* ROS-NF-кB activation.

### Oncogenic NRF2-HO-1 Activation Is Essential for DPP-4i-Driven ROS-Dependent NF-κB Activation in BC Cells

**Figure 4:**
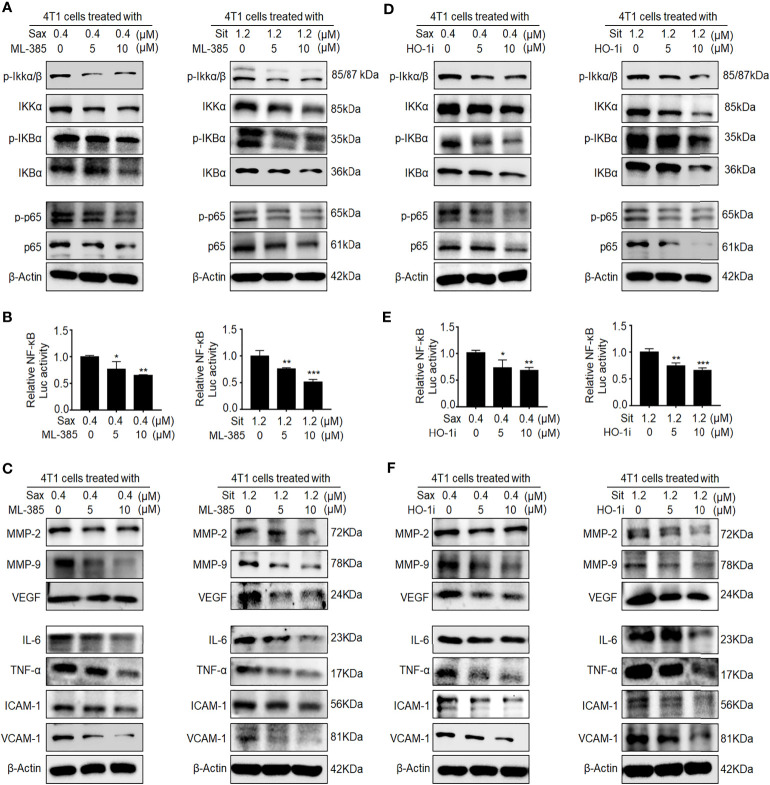
Inhibition of NRF2-HO-1 activation abrogates DPP-4i-driven NF-кB activation. **(A–C)** NRF2 blockage abrogates DPP-4i-induced NF-кB activation. 4T1 cells were co-treated with Sax (0.4 μM) or Sit (1.2 μM) and NRF2 inhibitor ML-385 (0, 5, and 10 μM), respectively. **(A)** The expression of p-65, p-p65, and NF-кB regulatory proteins was detected by Western blotting. **(B)** NF-кB transcriptional activation was analyzed by luciferase reporter assay, and **(C)** NF-кB-responsive targets were detected by Western blotting. **(D–F)** HO-1 inhibition attenuates DPP-4i-induced NF-кB activation. 4T1 cells were co-treated with Sax (0.4 μM) or Sit (1.2 μM) and HO-1 inhibitor (0, 5, and 10 μM), respectively. **(D)** The expression of p-65, p-p65, and NF-кB regulatory proteins was detected by Western blotting. **(E)** NF-кB transcriptional activation was analyzed by luciferase reporter gene assay. **(F)** NF-кB-responsive targets were detected by Western blotting. β-Actin was a loading control. Data are presented as mean ± SD of three independent experiments. Representative images are shown. *p < 0.05, **p < 0.01, and ***p < 0.001 between the indicated groups determined by the one-way analysis of variance (ANOVA).

Given the oncogenic roles of NRF2–HO-1 and NF-кB activations in DPP-4i-driven BC metastasis (7), we sought to explore the possible link between NRF2-HO-1 and NF-кB activations in DPP-4i-driven BC metastasis. First, we used NRF2 inhibitor ML-385 to investigate whether NRF2 could regulate NF-кB activation in DPP-4i-treated BC cells. We observed that ML-385-mediated NRF2 inhibition significantly reversed p-IKKα/β, IKKα, p-IKBα, and IKBα and p65 and p-p65 expressions (**Figure 4A**). Furthermore, NRF2 inhibition also attenuated DPP-4i-induced NF-кB transcriptional activation and NF-кB-responsive genes expression (**Figures 4B, C** **)**, suggesting that DPP-4i-driven NRF2 activation contributes to ROS-dependent NF-κB activation. Next, we applied HO-1 inhibitor to investigate the role of HO-1 activation in DPP-4i-driven NF-кB activation in BC cells. We observed that the HO-1 inhibitor significantly attenuated the DPP-4i-driven p-IKKα/β, IKKα, p-IKBα, and IKBα, and p65 and p-p65 expressions (**Figure 4D**). Furthermore, DPP-4i-driven NF-кB transcriptional activation and NF-кB-responsive targets expression were also markedly abrogated (**Figures 4E, F** **)**. Thus, these data indicate that NRF2-HO-1 activation contributes to ROS-mediated NF-кB activation in DPP-4i-treated BC cells *in vitro*.

**Figure 5:**
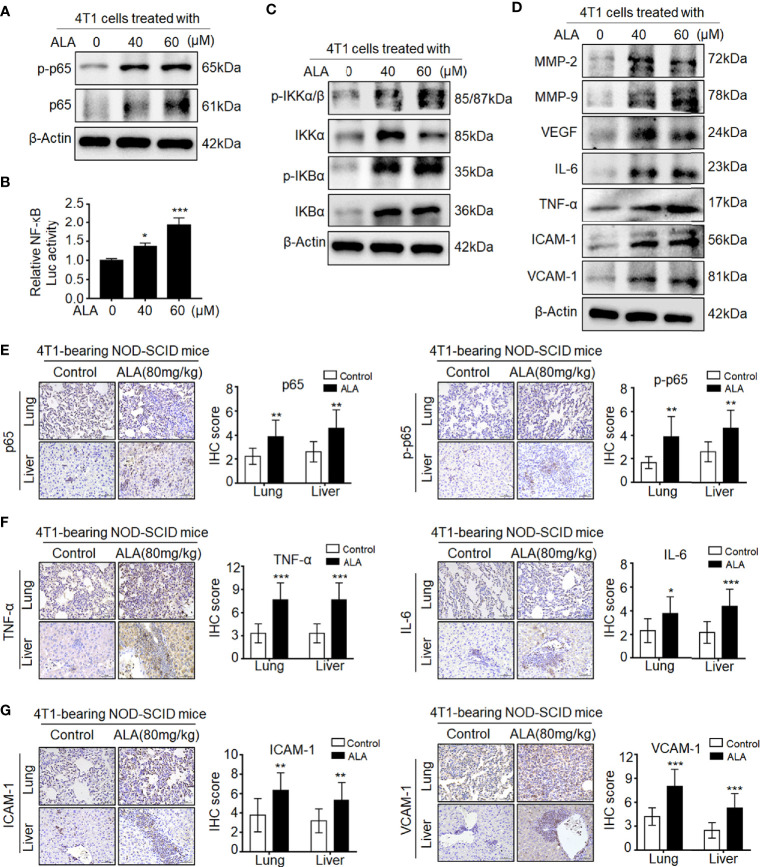
Pharmaceutical NRF2 activation promotes NF-кB activation *in vitro* and *in vivo*. **(A, B)** NRF2 activator ALA promotes NF-кB activation in BC cells. **(A)** 4T1 cells were treated with ALA (0, 40, and 60 μM), and p-65 and p-p65 expressions were detected by Western blotting. **(B)** NF-кB transcriptional activation was analyzed by luciferase reporter gene assay. **(C, D)** ALA enhances NF-кB-associated proteins expression *in vitro*. 4T1 cells were treated with ALA (0, 40, and 60 μM) for 4–6 h. **(C)** NF-кB regulatory proteins and **(D)** NF-кB-responsive targets were detected by Western blotting. β-Actin was a loading control. **(E–G)** ALA induces NF-кB activation and its downstream targets expression *in vivo*. 4T1-bearing NOD-SCID mice were treated with ALA (80 mg/kg) *via* intraperitoneal (i.p.) administration. IHC staining was used to detect **(E)** p-65 and p-p65, **(F)** IL-6 and TNF-α, and **(G)** ICAM-1 and VCAM-1 in lung and liver metastatic tissues, respectively. Data are presented as mean ± SD of three independent experiments. Scale bar: 50 μm. Representative images are shown. *p < 0.05, ** p < 0.01, and ***p < 0.001 between the indicated groups determined by unpaired Student’s t-test or the one-way analysis of variance (ANOVA).

Given the aberrant NRF2 activation in metastasis tissues of Sax-treated 4T1-bearing NOD-SCID mice (7), we investigated whether DPP-4i also could promote NRF2 activation in 4T1-bearing BALB/c mice. As shown in **Supplementary Figure S3**, we found that Sax or Sit treatment also enhanced NRF2-HO-1 activation in lung and liver metastasis tissues of 4T1-bearing BALB/c mice, suggesting that DPP-4i-induced NRF2-HO-1 activation is independent on immune status of 4T1-bearing mice model. Next, we used NRF2 activator ALA to test whether pharmaceutical NRF2 activation could promote NF-кB activation *in vitro and in vivo*. *In vitro*, we observed that ALA treatment significantly enhanced p65 and p-p65 levels and NF-кB transcriptional activation in BC cells (**Figures 5A, B** **)**. Meanwhile, p-IKKα/β, IKKα, p-IKBα, and IKBα, and NF-кB-responsive genes expressions were also increased upon ALA treatment *in vitro* (**Figures 5C, D** **)**. Moreover, in 4T1-bearing NOD-SCID mice, p65 and p-p65 expression and NF-кB-responsive protein levels were also enhanced after ALA treatment in lung and liver metastasis tissues (**Figures 5E–G**), indicating that pharmaceutical NRF2 activation promotes NF-кB activation *in vivo*. Together, these data suggest that NRF2–HO-1 activation plays a critical role in DPP-4i-driven ROS-dependent NF-kB activation of BC cells.

### DPP-4i Promotes the Recruitment of Tumor-Infiltrating Inflammatory and Immunosuppressive Cells in Metastatic Sites

**Figure 6:**
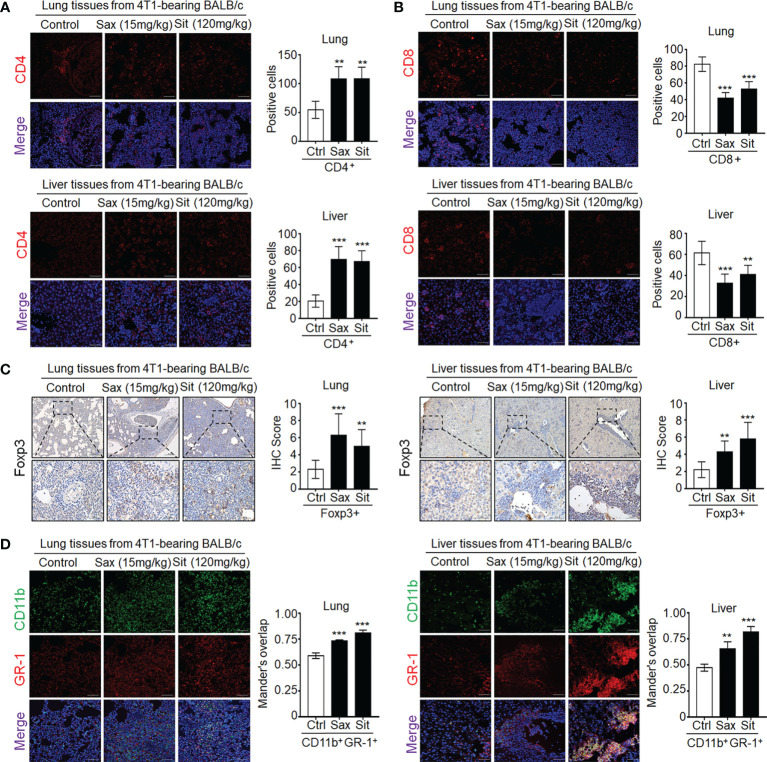
DPP-4i promotes tumor-infiltrating immune-suppressive cells in metastasis sites of BALB/c mice. **(A, B)** DPP-4i enhances CD4+ T cells but decreases CD8+ T cells in lung and liver metastatic tissues. 4T1-bearing BALB/c mice were treated with Sax (15 mg/kg) or Sit (120 mg/kg) *via* oral gavage daily, and **(A)** CD4+ T cells and **(B)** CD8+ T cells were detected by indirect IF staining in lung and liver metastatic tissues. **(C)** DPP-4i promotes Foxp3+ cells infiltration in metastatic tissues. Foxp3+ cells were detected by IHC staining in lung and liver metastatic tissues. **(D)** DPP-4i promotes the infiltration of CD11b^+^Gr-1^+^ MDSCs in metastatic tissues. CD11b and Gr-1 expressions were detected by direct IF double staining in lung and liver metastatic tissues. Nuclei were counterstained with DAPI. Data are presented as mean ± SD of three independent experiments. Representative images are shown. Scale bar: 50 μm. **p< 0.01, and ***p < 0.001 between the indicated groups determined by unpaired Student’s t-test or the one-way analysis of variance (ANOVA).

Given a critical role of tumor-infiltrating T cells in the prediction of clinical outcomes in BC patients (13), we further investigated the effect of DPP-4i on tumor-infiltrating immune cells in metastatic sites. Therefore, we analyzed the expression of immune-cell-associated markers in metastatic tissues including pan-leukocyte marker CD45, neutrophil marker MPO, macrophage markers CD11b, F4/80, and T cell markers CD4 and CD8 (14). We observed that DPP-4i significantly promoted the infiltration of CD45^+^, MPO^+^, and CD11b^+^/F4/80^+^ cells in lung and liver metastasis sites (**Supplementary Figure S4**). Furthermore, we also observed an increase in CD4^+^ cells but a decrease in CD8^+^ T cells in lung and liver metastasis sites (**Figures 6A, B** **)**, indicating that DPP-4i may induce tumor-immunosuppressive microenvironment in metastatic sites.

To further define whether DPP-4i is involved in the infiltration of immunosuppressive cells in metastatic sites (7–13), we further investigated the effect of DPP-4i on the infiltration of Treg cells and MDSCs in metastasis sites of 4T1-bearing BALB/c mice and observed a significant increase in tumor-infiltrating Foxp3^+^ cells in metastasis sites (**Figure 6C**), indicating that immunosuppressive Treg cells may be responsible for the increased tumor-infiltrating CD4^+^ T cells after DPP-4i treatment. Furthermore, we also observed that tumor-infiltrating MDSCs were also increased in lung and liver metastasis tissues of DPP-4i-treated mice (**Figure 6D**), indicating that DPP-4i may promote 4T1-induced recruitment or expansion of immunosuppressive cells in metastasis tissues.

Given MDSCs as a major immunosuppressive population in 4T1-bearing BALB/c mice (13, 20), we analyzed the effect of DPP-4i on MDSCs proliferation and differentiation in PBMCs and splenic cells of 4T1-bearing BALB/c mice. Interestingly, we did not observe an obvious increase in CD11b^+^GR-1^+^ MDSCs in PBMCs or splenic cells (**Supplementary Figure S5A**). Then, we evaluated MDSCs differentiation by analyzing the percentage of G-MDSCs and Mo-MDSCs, two major subtypes of MDSCs, but did not find a significant alteration of MDSCs differentiation in PBMCs and splenic cells of 4T1-bearing mice (**Supplementary Figure S5B**), indicating that therapeutic ranges of DPP-4i may not exert direct effects on biologic behavior of MDSCs *in vivo*. To obtain more direct evidence, next, we set up an *in vitro* co-culture system in which DPP-4i was cultured with 4T1-induced splenic MDSCs. However, MDSCs differentiation, cell viability, and cell apoptosis were not markedly changed after DPP-4i treatment (**Supplementary Figures S5C** and **S6A, B**). In addition, DPP-4i treatment did not significantly promote ROS release, NRF2 activation, and expression of suppressive molecules including ARG-1 (Arginase-1), NCF1 (NOX components P47^phox^), CYBB (NOX components gp91^phox^), TGF-β, and IL-10 in 4T1-induced splenic MDSCs (**Supplementary Figures S6C–E**). Thus, these data suggest that DPP-4i may induce tumor immunosuppressive microenvironment by promoting recruitment or expansion of tumor-infiltrating Treg and (or) MDSCs *via* an indirect manner.

### DPP-4i Reprograms Tumor Microenvironment by Direct Interaction With BC Cells *via* ROS–NF-кB–NLRP3 Axis

**Figure 7:**
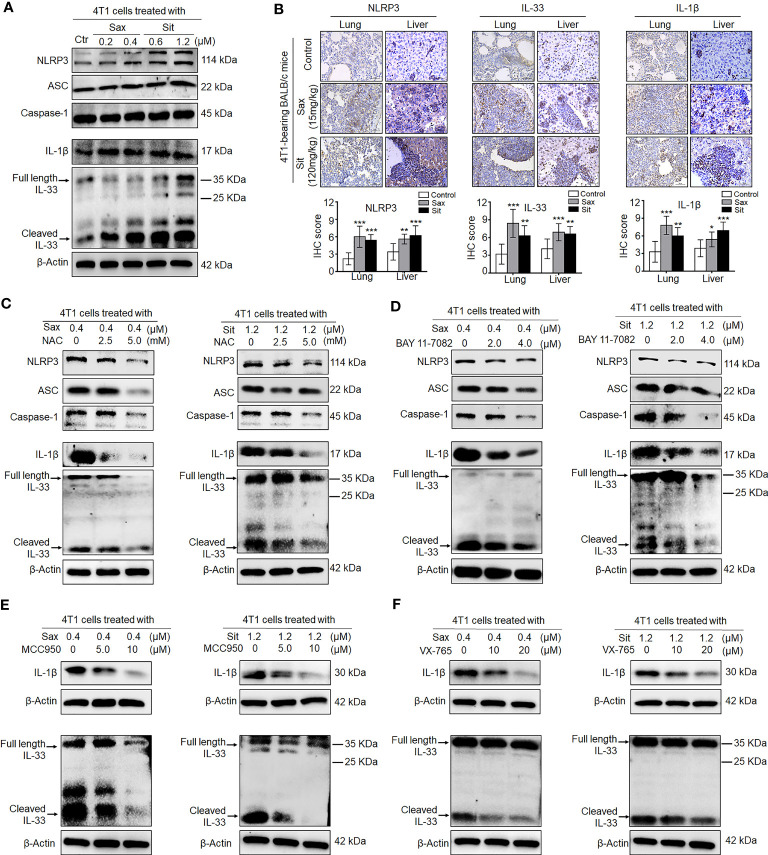
DPP-4i triggers NLRP3 inflammasome activation *via* ROS–NF-кB axis. **(A)** DPP-4i triggers NLRP3 inflammasome activation in 4T1 cells. 4T1 cells were treated with Sax (0.4 μM) or Sit (1.2 μM) for 24 h, and NLRP3 inflammasome-associated proteins were detected by Western blotting. **(B)** DPP-4i enhances NLRP3 inflammasome of BC cells *in vivo*. 4T1-bearing BALB/c mice were treated with Sax (15 mg/kg) or Sit (120 mg/kg) *via* oral gavage daily. NLRP3, IL-1β, and IL-33 were detected by IHC staining in lung and liver metastatic tissues. **(C)** ROS inhibition attenuates DPP-4i-induced NLRP3 inflammasome activation. 4T1 cells were co-treated with Sax (0.4 μM) or Sit (1.2 μM) and NAC (0, 2.5, and 5 mM), respectively, and NLRP3 inflammasome- associated proteins were detected by Western blotting. **(D)** NF-кB blockage decreases DPP-4i-triggered NLRP3 inflammasome activation. 4T1 cells were co-treated with Sax (0.4 μM) or Sit (1.2 μM) and BAY 11-7082 (0, 2, and 4 µM) respectively, and NLRP3 inflammasome-associated proteins were detected by Western blotting. **(E)** Inhibition of NLRP3 activation attenuates DPP-4i-triggered IL-1β and IL-33 expression. 4T1 cells were co-treated with Sax (0.4 μM) or Sit (1.2 μM) and NLRP3 inhibitor MCC950 (0, 5, and 10 μM) respectively, and IL-1β and IL-33 cytokines were detected by Western blotting. **(F)** Inhibition of caspase-1 activation attenuates DPP-4i-triggered IL-1β and IL-33 expression. 4T1 cells were cotreated with Sax (0.4 μM) or Sit (1.2 μM) and caspase-1 inhibitor XV-765 (0, 10, and 20 µM), respectively, and IL-1β and IL-33 cytokines were detected by Western blotting. β-Actin was a loading control. Data are presented as mean ± SD of three independent experiments. Representative images are shown. Scale bar: 50 μm. *p < 0.05, **p < 0.01, and ***p < 0.001 between the indicated groups determined by unpaired Student’s t-test or the one-way analysis of variance (ANOVA).

Given an indirect role of DPP-4i in the remodeling of tumor immunosuppressive microenvironment, we sought to know whether DPP-4i could orchestrate tumor microenvironment by direct interaction with BC cells. To this end, we first investigated the effect of DPP-4i on NLRP3 activation, a critical inflammasome in the remodeling of tumor microenvironment (21, 22). We observed that DPP-4i obviously promoted NLRP3 inflammasome activation and IL-1β and IL-33 expressions *in vitro* and 4T1-bearing BALB/c mice (**Figures 7A, B** **)**. Similar results were also observed in Sax-treated 4T1-bearing NOD-SCID mice (**Supplementary Figure S7**). However, we did not find that inhibition of NLRP3 inflammasome by MCC950 can inhibit DPP-4i-driven BC cell migration and invasion *in vitro* (**Supplementary Figure S8**), indicating that NLRP3 inflammasome may not be directly involved in BC metastasis *in vitro*. Thus, these data suggest that DPP-4i can trigger NLRP3 inflammasome activation by direct interaction with BC cells, thereby contributing to the remodeling of tumor microenvironment.

Given the aberrant activation of ROS–NRF2–HO-1–NF-кB axis in DPP-4i-treated BC cells, we explored potential roles of ROS–NRF2–HO-1–NF-кB axis in DPP-4i-driven NLRP3 inflammasome activation in BC cells. First, we investigated the effect of NRF2 activation on NLRP3 activation. As shown in **Supplementary Figure S9**, NRF2 activator ALA significantly enhanced NLRP3 inflammasome activation and IL-1β and IL-33 levels *in vitro* and in 4T1-bearing NOD-SCID mice, indicating a vital role of NRF2 activation in DPP-4i-driven NLRP3 activation. Then, we used ROS or NF-кB inhibitors to investigate the role of ROS-NF-кB activation in DPP-4i-driven NLRP3 activation and also found that both ROS and NF-кB inhibitions can significantly abrogate DPP-4i-driven NLRP3 inflammasome activation and IL-1β and IL-33 levels (**Figures 7C, D** **)**, indicating that DPP-4i-driven NLRP3 activation is ROS–NF-кB dependent. Moreover, we explored the role of NLRP3 activation in the expression of IL-1β and IL-33 cytokines and found that NLRP3 inhibitor MCC950 and caspase-1 inhibitor VX-765 significantly attenuated DPP-4i-driven IL-1β and IL-33 levels (**Figures 7E, F** **)**, suggesting that DPP-4i triggers ROS–NF-кB–NLRP3 activation, leading to caspase-1-mediated processing of IL-1β and IL-33.

**Figure 8:**
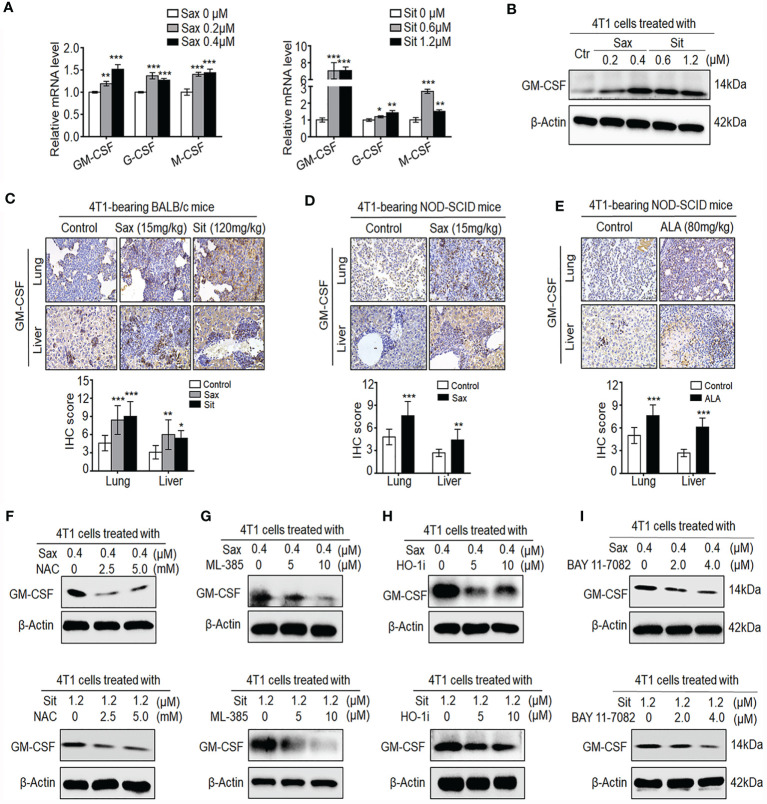
DPP-4i promotes the secretion of GM-CSF, G-CSF, and M-CSF *via* ROS–NRF2–HO-1-NF-кB axis in BC cells. **(A, B)** DPP-4i increases GM-CSF, G-CSF, and M-CSF secretion in 4T1 cells. 4T1 cells were treated with Sax (0, 0.2, and 0.4 μM) or Sit (0, 0.6, and 1.2 μM) for 24 h. **(A)** mRNA level of GM-CSF, G-CSF, and M-CSF was analyzed by qRT-PCR, and **(B)** GM-CSF protein was detected by Western blotting. **(C)** DPP-4i promotes GM-CSF secretion of 4T1 cells *in vivo*. 4T1-bearing BALB/c mice were treated with Sax (15 mg/kg) or Sit (120 mg/kg) *via* oral gavage daily. GM-CSF were detected in lung and liver metastatic tissues by IHC staining. **(D, E)** 4T1-bearing NOD-SCID mice were treated with Sax (15 mg/kg) *via* **(D)** oral gavage daily or ALA (80 mg/kg) *via* **(E)** intraperitoneal (i.p.) administration, and GM-CSF expression was detected in lung and liver metastatic tissues by IHC staining. **(F)** ROS inhibition attenuates DPP-4i-induced GM-CSF secretion. 4T1 cells were co-treated with Sax (0.4 μM) or Sit (1.2 μM) and NAC (0, 2.5, and 5 mM), respectively, and GM-CSF expression was detected by Western blotting. **(G, H)** Inhibition of NRF2-HO-1 abrogates DPP-4i-induced GM-CSF secretion. 4T1 cells were co-treated with Sax (0.4 μM) or Sit (1.2 μM), and **(G)** ML-385 (0, 5, and 10 μM) or **(H)** HO-1 inhibitor (0, 5, and 10 μM) respectively. Then, GM-CSF expression was detected by Western blotting. **(I)** NF-кB blockage abrogates DPP-4i-induced GM-CSF secretion. 4T1 cells were co-treated with Sax (0.4 μM) or Sit (1.2 μM) and BAY 11-7082 (0, 2, and 4 μM), respectively, and GM-CSF expression was detected by Western blotting. β-Actin was used as a loading control. Representative images are shown. Data are presented as mean ± SD of three independent experiments. Scale bar: 50 μm. *p < 0.05, **p < 0.01, and ***p < 0.001 between the indicated groups determined by unpaired Student’s t-test or the one-way analysis of variance (ANOVA).

It has been shown that GM-CSF, G-CSF, and M-CSF cytokines can induce accumulation and expansion of MDSCs, leading to the enhancement of the 4T1 BC metastasis (20, 23). Thus, we further investigated whether these cytokines were involved in DPP-4i-induced tumor immunosuppressive microenvironment in BC cells. As shown in **Figure 8A**, we observed that both Sax or Sit treatment markedly promoted transcription levels of G-CSF, M-CSF, and GM-CSF in BC cells, suggesting that DPP-4i may directly induce G-CSF, M-CSF, and GM-CSF secretion in BC cells. Given the essential role of GM-CSF in the recruitment and maintenance of MDSCs of tumor-immunosuppressive microenvironment (24), we then focused on how DPP-4i can regulate GM-CSF expression *in vitro* and *in vivo*. Using Western blotting and IHC staining, we found that DPP-4i significantly upregulated GM-CSF expression *in vitro* and in 4T1-bearing BALB/c mice (**Figures 8B, C** **)**. Meanwhile, in 4T1-bearing NOD-SCID mice, we also observed that Sax or ALA treatments also enhanced GM-CSF levels in lung and liver metastasis tissues (**Figures 8D, E** **)**, indicating that ROS–NRF2–HO-1–NF-кB axis play a crucial role in DPP-4i-driven GM-CSF secretion in BC cells. To further verify these results, we used a serial of chemical inhibitors to investigate whether ROS–NRF2–HO-1–NF-кB inhibition could reverse DPP-4i-driven GM-CSF secretion in BC cells. We found that no matter the ROS–NRF2–HO-1 inhibition or NF-кB inhibition, it significantly attenuated DPP-4i-driven GM-CSF expression in BC cells (**Figures 8F–I**), suggesting an essential role of ROS–NRF2–HO-1–NF-кB axis in DPP-4i-driven GM-CSF production in BC cells. Together, these results suggest that DPP-4i reprograms tumor microenvironment by interaction with BC cells *via* the ROS–NF-кB–NLRP3 axis.

## Discussion

Better understanding the role of antidiabetic DPP-4i in BC-induced tumor microenvironment would not only offer novel insights into its potential role in BC progression but also may provide new strategies to alleviate the dark side of DPP-4i in diabetic patients with BC. Here, our results presented a novel finding that DPP-4i can reprogram tumor microenvironment that facilitates murine breast cancer metastasis by interacting with cancer cells *via* a ROS–NRF2–HO-1–NF-кB–NLRP3 axis, providing an immune mechanistic insight into the dark side of DPP-4i in BC progression.

Our finding demonstrates that DPP-4i promotes BC metastasis by triggering NF-кB activation *via* a ROS–NRF2–HO-1-dependent manner, offering more mechanistic insights into the oncogenic role of ROS–NRF2–HO-1 axis in DPP-4i-driven BC metastasis. Our recent finding reveals that DPP-4i can facilitate murine BC metastasis by oncogenic ROS–NRF2–HO-1 axis *via* a positive NRF2–HO-1 feedback loop (7). However, the downstream signaling underlying ROS–NRF2–HO-1 axis mediates DPP-4i-induced BC metastasis has not yet been completely elucidated. Of note, we noted that ROS–NRF2–HO-1 axis promoted DPP-4i-induced MMP-2, MMP-9, and VEGF levels (7), three well-known NF-кB-responsive targets (14), promoting us to investigate whether NF-кB activation is involved in DPP-4i-induced BC metastasis. Here, our data showed that DPP-4i triggered aberrant NF-кB activation in both immune-deficient NOD-SCID and immune-competent BALB/C mice. Subsequently, we also revealed that ROS-NF-кB inhibition abrogated DPP-4i-driven BC metastasis, while abrogation of NRF2-HO-1 attenuated DPP-4i-driven ROS-dependent NF-kB activation in BC cells. Moreover, pharmaceutical NRF2 activation by ALA also promoted NF-кB activation *in vitro* and in 4T1-bearing NOD-SCID mice. Thus, our results strongly suggest that aberrant NF-кB activation, as a downstream signaling of ROS–NRF2–HO-1 axis, plays an essential role in DPP-4i-driven BC metastasis, further improving our understanding of the role of DPP-4i in the BC progression. However, the regulation of DPP-4i-driven NRF2 to NF-кB activation has not been completely demonstrated in 4T1 cells, and further study is need to dissect the more mechanistic details.

Our present finding reveals that DPP-4i promotes tumor-infiltrating inflammation and immune-suppressive cells in metastatic sites, offering new strategies to develop effective immunotherapeutic approaches to alleviate DPP-4i-driven BC metastasis. A previous report suggested a potential role of DPP-4 inhibition in CXCL10-mediated lymphocyte trafficking in melanoma B16F10-bearing mice (5). However, tumor-infiltrating T cells (TILs) rather than circulating T cells were shown to play a critical role in the prediction of clinical outcomes of BC patients (13), promoting us to focus on tumor-infiltrating immune cells in metastatic tissues. 4T1 cells originally from BALB/c mice share many characteristics with naturally occurring human BC and can metastasize to distant lung and liver organs, providing an ideal mice model for mimicking the metastatic and advanced stages of human BC (13). MDSCs, as a major immunosuppressive population in 4T1-bearing BALB/c mice (13, 20), contribute to tumor-immunosuppressive microenvironment not only by producing a serial of suppressive molecules such as Arg-1, NCF1, CYBB, TGF-β, and IL-10 (25, 26) but also by promoting recruitment and expansion of Treg cells *via* TGF-β and IL-10 (26). Our current finding revealed that DPP-4i enhanced tumor-infiltrating MPO^+^, CD4^+^, F4/80^+^, Foxp3^+^ cells, and MDSCs, but decreased CD8^+^ T lymphocytes in metastatic sites, indicating that DPP-4i may induce tumor-immunosuppressive microenvironment by enhancing tumor-infiltrating immune-suppressive cells. However, our current finding showed no direct effects of DPP-4i on cell viability, apoptosis, differentiation, and even immune-suppressive molecule levels in 4T1-induced PBMC or splenic MDSCs. Thus, these findings indicate that DPP-4i may induce the recruitment or expansion of tumor-infiltrating MDSCs *via* an indirect mechanism.

Our finding further highlights that DPP-4i as a potential orchestrator may contribute to the tumor-immune-suppressive microenvironment by direct interaction with BC cells *via* ROS–NF-кB–NLRP3 axis, providing more immune mechanistic insights into the DPP-4i-driven infiltration of immunosuppressive cells in BC metastasis. Our finding suggest an indirect role of DPP-4i in the recruitment of tumor-infiltrating MDSCs, raising a possibility that DPP-4i may reprogram tumor microenvironment by direct interaction with BC cells, thereby promoting infiltration of immune-suppressive cells in metastatic sites. Our previous finding revealed that NF-кB inhibition in human gastric cancer cells inhibited tumor-infiltrating CD11c, F4/80, CD11b, and Gr-1-positive cells in lung and liver metastatic tissues of BALB/c nude (nu/nu) mice (14), indicating a critical role of NF-кB activation in the remodeling of tumor microenvironment. Here, our finding revealed that DPP-4i-induced NF-кB activation not only enhanced metastasis-associated MMP-2, MMP-9, IL-6, and VEGF levels but also increased adhesion proteins ICAM-1 and VCAM-1. Noteworthy, among these downstream targets, VEGF was reported to induce inflammatory neovascularization for pathological hemangiogenesis and lymphangiogenesis by recruiting inflammation monocytes and (or) macrophages (14, 23), while ICAM-1 and VCAM-1, as ligands by LFA-1 and Mac-1 (CD11b) expressed in leukocytes, were also shown to contribute to the recruitment of circulating leukocytes into the inflammation sites (14, 21, 23). Furthermore, our finding also showed that DPP-4i promoted NF-кB-dependent secretion of G-CSF, M-CSF, and GM-CSF, three well-known cytokines for the accumulation and expansion of MDSCs (20, 23), while a combination of GM-CSF/IL-6 or other cytokines such as TGF-β and VEGF was also shown to induce immune-suppressive MDSCs (25, 27). Therefore, these data suggest that DPP-4i may contribute to the modification of tumor microenvironment by releasing a serial of adhesion or cytokines *via* NF-кB activation in BC cells.

More significantly, our finding revealed that DPP-4i also triggered NF-кB-dependent NLRP3 inflammasome activation, leading to caspase-1-mediated processing of IL-1β and IL-33, two critical proinflammatory cytokines for the tumor-immune-suppressive microenvironment (21, 22). IL-1β has been shown to facilitate BC tumor metastasis by multiple routes, including modulating the immune cell milieu, promoting the recruitment of MDSCs, and increasing adhesion molecules levels at the metastasis sites (21). While IL-33, a novel member of the IL-1 family of cytokines, also plays a critical role in the modulation of the metastatic immune microenvironment by facilitating intratumoral accumulation of immunosuppressive and innate lymphoid cells (22, 28, 29). Here, our finding showed that DPP-4i promoted caspase-1-dependent processing of IL-1β and IL-33 by activating NF-кB–NLRP3 activation, indicating that DPP-4i may reprogram tumor microenvironment by promoting 4T1 cells-derived IL-1β and IL-33 *via* NF-кB–NLRP3 pathway. However, due to complex cell types or cytokines in tumor microenvironment, besides MDSCs, our present finding has not completely demonstrated the effect of DPP-4i on the development of others tumor-immunosuppressive cells like Treg cells, which should be further clarified in ongoing study. Overall, these results suggest that DPP-4i can reprogram tumor microenvironment by direct interaction with BC cells *via* ROS–NF-кB–NLRP3 axis, offering novel insights relevant for the development of effective immunotherapeutic approaches to alleviate DPP-4i-driven BC metastasis.

In summary, our study suggests that antidiabetic DPP-4i as a potential orchestrator reprograms tumor microenvironment that facilitates murine BC metastasis by interacting with BC cells *via* a ROS–NRF2–HO-1–NF-κB–NLRP3 axis. This finding not only provides a mechanistic insight into the oncogenic role of ROS–NRF2–HO-1 in DPP-4i-driven BC progression but also offers novel insights relevant for the development of effective immunotherapeutic approaches to alleviate the dark side of DPP-4i in BC progress.

## Data Availability Statement

The original contributions presented in the study are included in the article/**Supplementary Material**. Further inquiries can be directed to the corresponding author.

## Ethics Statement

The animal study was reviewed and approved by Institutional Animal Care and Use Committee of Children’s Hospital of Chongqing Medical University.

## Author Contributions

Conception and design: RL, XZ, MY, and YH. Development of methodology: RL, XZ, TY, XW, BX, and XX. Acquisition of data (provided animals, provided facilities, etc.): RL, XZ, TY, XW, BX, and XX. Analysis and interpretation of data (e.g., statistical analysis): RL, XZ, JF, and YH. Writing, review, and/or revision of the manuscript: RL, XZ, MY, LB, and YH. Administrative, technical, or material support (i.e., reporting or organizing data, constructing databases): RL, XX, JF, XW, and BX. Study supervision: YH. All authors contributed to the article and approved the submitted version.

## Funding

This study was partly supported by the National Natural Science Foundation of China (No. 32171119, to YH), Chongqing basic and frontier research project (CSTC2018jcyjAX0218, to YH) and Chongqing Yuzhong District Sci & Tech Research Project (20190106, to YH).

## Conflict of Interest

The authors declare that the research was conducted in the absence of any commercial or financial relationships that could be construed as a potential conflict of interest.

## Publisher’s Note

All claims expressed in this article are solely those of the authors and do not necessarily represent those of their affiliated organizations, or those of the publisher, the editors and the reviewers. Any product that may be evaluated in this article, or claim that may be made by its manufacturer, is not guaranteed or endorsed by the publisher.

## Supplementary Material

Supplementary Figure S1 Sax induces aberrant NF-кB activation in NOD-SCID mice. 4T1-bearing NOD-SCID mice were treated with or without Sax (15 mg/kg) *via* oral gavage daily. IHC staining was performed to detect p65 **(A)**, p-p65 **(B)** and NF-кB-responsive proteins **(C–F)** in lung and liver metastatic tissues. Data are presented as mean ± SD of three independent experiments. Representative images are shown. Scale bar: 50 μm. *p < 0.05, **p < 0.01 and ***p < 0.001 between the indicated groups determined by unpaired student’s t-test.

Supplementary Figure S2 ROS inhibition reverses DPP-4i-driven cell migration and invasion *in vitro*. 4T1 cells were subject to cell migration **(A)** and cell invasion **(B)** assays upon co-treatment with Sax (0.4 μM) or Sit (1.2 μM) and NAC (0, 2.5 mM, 5 mM) for 24h respectively. Migration or invasion cells were counted in 5-10 random fields (200×magnification). Data are presented as mean ± SD of three independent experiments. Representative images are shown. *p < 0.05, **p < 0.01 and ***p < 0.001 between the indicated groups determined by the one-way analysis of variance (ANOVA).

Supplementary Figure S3 DPP-4i induces aberrant NRF2-HO-1 activation in BALB/c mice. 4T1-bearing BALB/c mice were treated with Sax (15 mg/kg) or Sit (120 mg/kg) *via* oral gavage daily respectively. IHC staining was performed to detect p-NRF2 **(A)**, NRF2 **(B)** and NRF2-responsive HO-1 **(C)** expression in lung and liver metastatic tissues. Data are presented as mean ± SD of three independent experiments. Representative images are shown. Scale bar: 50 μm. *p < 0.05, **p < 0.01 and ***p < 0.001 between the indicated groups determined by the one-way analysis of variance (ANOVA).

Supplementary Figure S4 DPP-4i promotes infiltration of CD45, MPO and F4/80-positive cells in metastatic sites of 4T1-bearing BALB/c mice. 4T1-bearing BALB/c mice were treated with Sax (15 mg/kg) or Sit (120 mg/kg) *via* oral gavage daily. CD45 **(A)** and MPO **(B)** expression was detected by indirect IF staining, and CD11b/F4/80 **(C)** expression was detected by direct IF double staining in lung and liver metastatic tissues. Nuclei were counterstained with DAPI. Data are presented as mean ± SD of three independent experiments. Representative images are shown. Scale bar:50 μm. *p < 0.05, **p < 0.01 and ***p < 0.001 between the indicated groups determined by the one-way analysis of variance (ANOVA).

Supplementary Figure S5 DPP-4i does not alter MDSCs differentiation *in vitro* and *in vivo*. **(A, B)** DPP-4i does not alter MDSCs differentiation *in vivo*. 4T1-bearing BALB/c mice were treated with Sax (15 mg/kg) or Sit (120 mg/kg) *via* oral gavage daily. Cell percentage of CD11b^+^Gr-1^+^ MDSCs in PBMCs or Splenic cells was evaluated by flow cytometry **(A)**. Cell percentage of Mo-MDSCs (Ly6C^hi^Ly6G^-^) and G-MDSCs (Ly6C^lo^Ly6G^+^) in PBMCs or splenic cells was evaluated by gating on CD11b^+^ population using flow cytometry **(B)**. **(C)** DPP-4i does not alter MDSCs differentiation *in vitro*. CD11b^+^Gr-1^+^ MDSCs from splenic cells of 4T1-bearing BALB/c mice were co-cultured with Sax (0, 0.2 μM, 0.4 μM) or Sit (0, 0.6 μM, 1.2 μM) in the presence of GM-CSF (10 ng/ml) for 72-96h. MDSCs differentiation was evaluated by analyzing Mo-MDSCs and G-MDSCs using flow cytometry. Data are presented as mean ± SD of three independent experiments. *p < 0.05, **p < 0.01 and ***p < 0.001 between the indicated groups determined by the one-way analysis of variance (ANOVA).

Supplementary Figure S6 DPP-4i treatment does not alter cell viability, apoptosis, ROS production, NRF2 activation and immune-suppressive molecules in MDSCs *in vitro*. CD11b^+^Gr-1^+^ MDSCs isolated from splenic cells of 4T1-bearing BALB/c mice were co-cultured with Sax (0, 0.2 μM, 0.4 μM) or Sit (0, 0.6 μM, 1.2 μM) in the presence of GM-CSF (10 ng/ml) for 24-96 h. Cell viability (co-cultured for 24-96 h) was detected by CCK-8 assay **(A)** and cell apoptosis (co-cultured for 72-96 h) was detected by Annexin-V-PE/7-AAD staining **(B)**, respectively. Intracellular ROS and mROS in DPP-4i-treated MDSCs were detected using DHE and MitoSox staining by flow cytometry **(C)** respectively. The expressions of NRF2-responsive genes **(D)** and immune-suppressive genes **(E)** were detected by qRT-PCR. β-actin was as an internal control. Data are presented as mean ± SD of three independent experiments. *p < 0.05, **p < 0.01 and ***p < 0.001 between the indicated groups determined by the one-way analysis of variance (ANOVA).

Supplementary Figure S7 Sax triggers NLRP3 activation of BC cells in NOD-SCID mice. 4T1-bearing NOD-SCID mice were treated with Sax (15 mg/kg) *via* oral gavage daily. IHC staining was performed to detect NLRP3 **(A)**, IL-33 **(B)** and IL-1β **(C)** expression in lung and liver metastatic tissues. Data are presented as mean ± SD of three independent experiments. Representative images are shown. Scale bar: 50 μm. *p < 0.05, **p < 0.01 and ***p < 0.001 between the indicated groups determined by unpaired student’s t-test.

Supplementary Figure S8 Inhibition of NLRP3 activation do not suppress BC migration and invasion *in vitro*. 4T1 cells were subject to cell migration **(A)** and cell invasion **(B)** analysis upon co-treatment of Sax (0.4 μM) or Sit (1.2 μM) and NLRP3 inhibitor MCC950 (0, 5 μM,10 μM) for 24h respectively. Migration or invasion cells were counted in 5-10 random fields (200×magnification). Data are presented as mean ± SD of three independent experiments. Representative images are shown. *p < 0.05, **p < 0.01 and ***p < 0.001 between the indicated groups determined by the one-way analysis of variance (ANOVA).

Supplementary Figure S9 ALA promotes NLRP3 inflammasome activation of BC cells *in vitro* and *in vivo*. **(A)** NRF2 activator ALA induces NLRP3 inflammasome activation *in vitro*. 4T1 cells were treated with ALA (0, 40 μM, 60 μM) for 4-6 h, and NLRP3 inflammasome-associated proteins were detected by western blotting. β-actin was a loading control. **(B)** ALA enhances NLRP3 inflammasome of BC cells *in vivo*. 4T1-bearing NOD-SCID mice were treated with or without ALA (80 mg/kg) *via* intraperitoneal (i.p.) administration. NLRP3, IL-1β and IL-33 expressions were detected by IHC staining in lung and liver metastatic tissues. Data are presented as mean ± SD of three independent experiments. Scale bar: 50 μm. *p < 0.05, **p < 0.01 and ***p < 0.001 between the indicated groups determined by unpaired student’s t-test.
