# Supplementary figures and images for: Antidiabetic DPP-4 Inhibitors Reprogram Tumor Microenvironment That Facilitates Murine Breast Cancer Metastasis Through Interaction With Cancer Cells via a ROS–NF-кB–NLRP3 Axis
Source: Front Oncol. 2021 Sep 24;11:728047. doi: 10.3389/fonc.2021.728047 (PMC8497989; doi:10.3389/fonc.2021.728047)

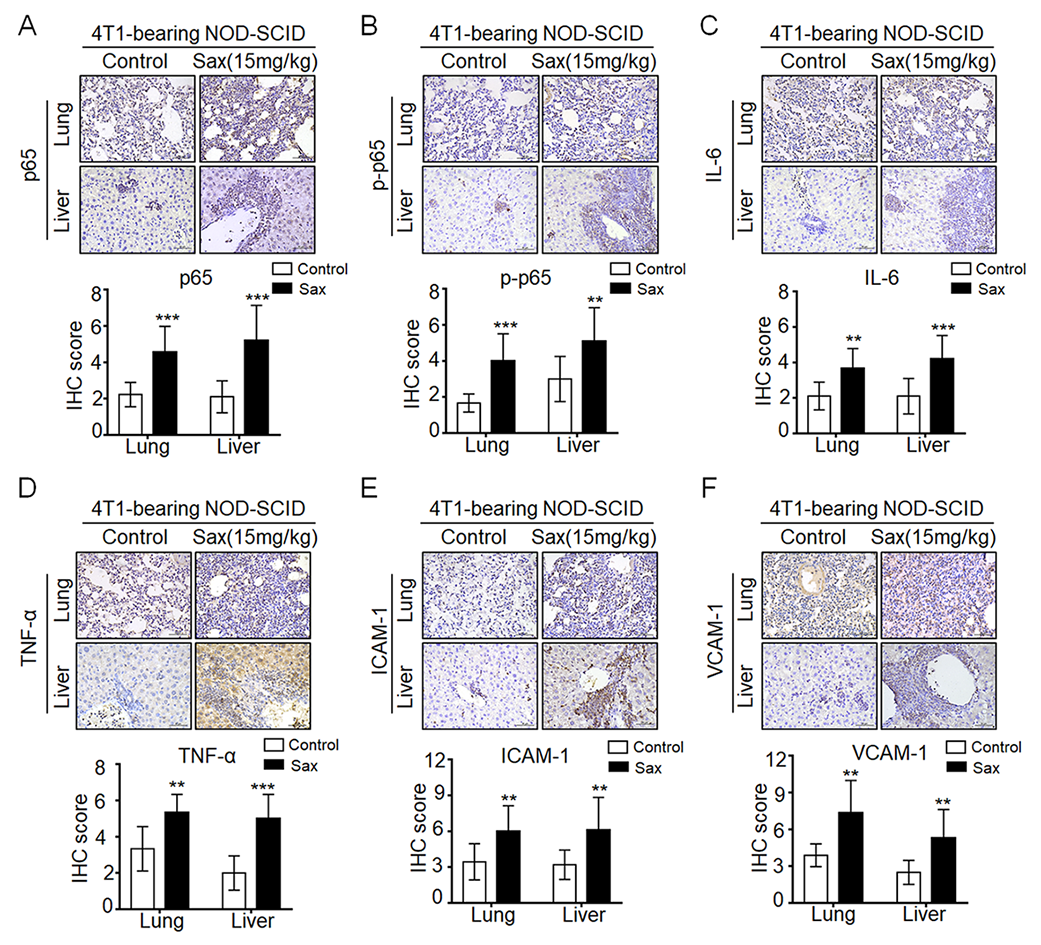

Supplement: Supplementary Figure S1 — Sax induces aberrant NF-кB activation in NOD-SCID mice. 4T1-bearing NOD-SCID mice were treated with or without Sax (15 mg/kg) via oral gavage daily. IHC staining was performed to detect p65 (A), p-p65 (B) and NF-кB-responsive proteins (C–F) in lung and liver metastatic tissues. Data are presented as mean ± SD of three independent experiments. Representative images are shown. Scale bar: 50 μm. *p < 0.05, **p < 0.01 and ***p < 0.001 between the indicated groups determined by unpaired student’s t-test. [file Image_1.tif]

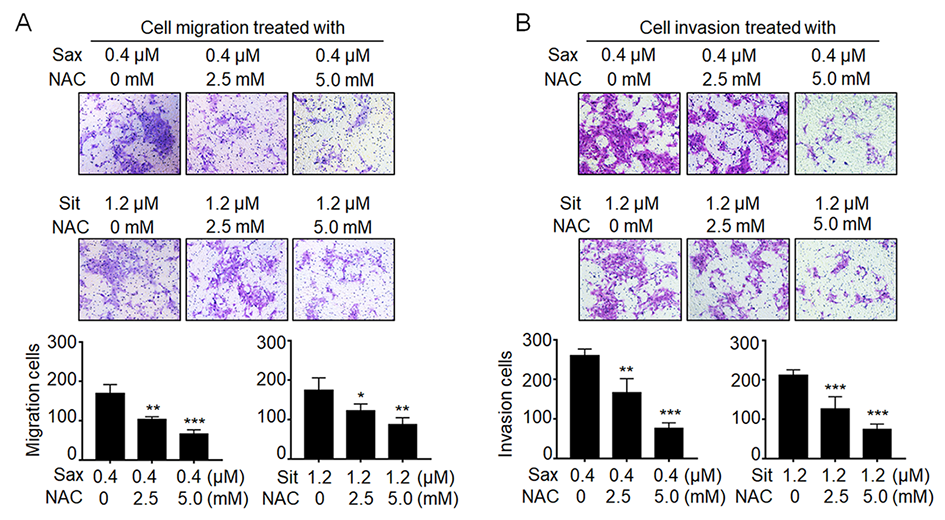

Supplement: Supplementary Figure S2 — ROS inhibition reverses DPP-4i-driven cell migration and invasion in vitro. 4T1 cells were subject to cell migration (A) and cell invasion (B) assays upon co-treatment with Sax (0.4 μM) or Sit (1.2 μM) and NAC (0, 2.5 mM, 5 mM) for 24h respectively. Migration or invasion cells were counted in 5-10 random fields (200×magnification). Data are presented as mean ± SD of three independent experiments. Representative images are shown. *p < 0.05, **p < 0.01 and ***p < 0.001 between the indicated groups determined by the one-way analysis of variance (ANOVA). [file Image_2.tif]

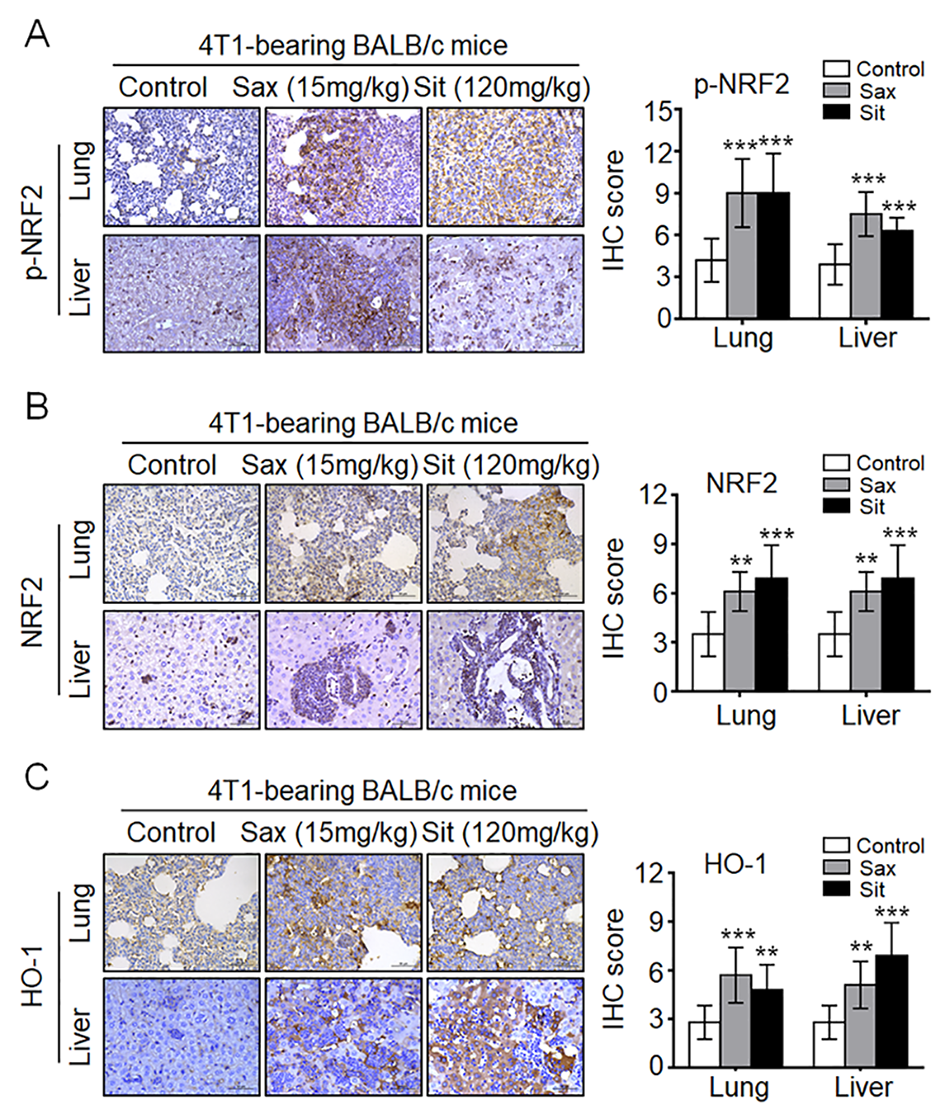

Supplement: Supplementary Figure S3 — DPP-4i induces aberrant NRF2-HO-1 activation in BALB/c mice. 4T1-bearing BALB/c mice were treated with Sax (15 mg/kg) or Sit (120 mg/kg) via oral gavage daily respectively. IHC staining was performed to detect p-NRF2 (A), NRF2 (B) and NRF2-responsive HO-1 (C) expression in lung and liver metastatic tissues. Data are presented as mean ± SD of three independent experiments. Representative images are shown. Scale bar: 50 μm. *p < 0.05, **p < 0.01 and ***p < 0.001 between the indicated groups determined by the one-way analysis of variance (ANOVA). [file Image_3.tif]

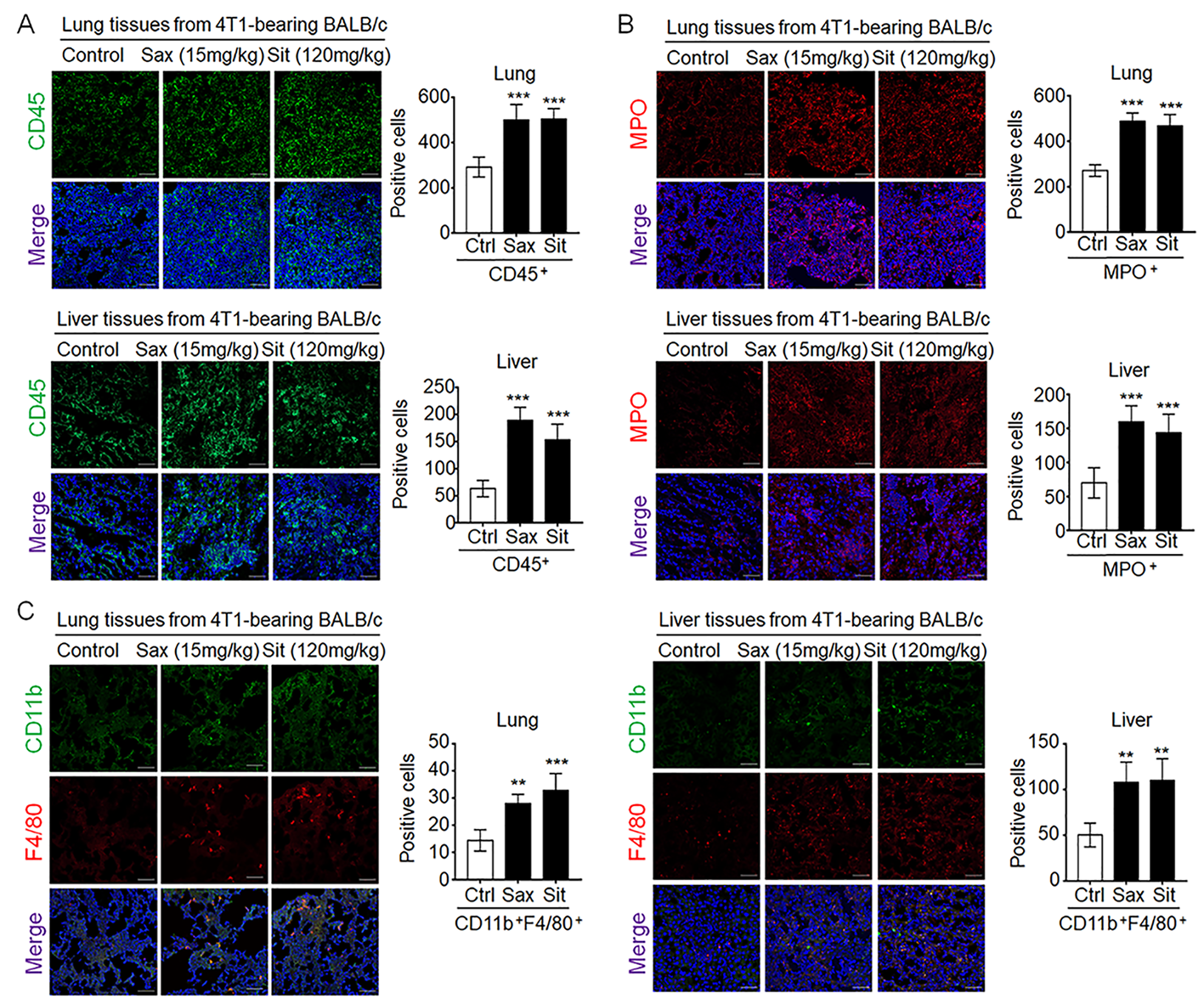

Supplement: Supplementary Figure S4 — DPP-4i promotes infiltration of CD45, MPO and F4/80-positive cells in metastatic sites of 4T1-bearing BALB/c mice. 4T1-bearing BALB/c mice were treated with Sax (15 mg/kg) or Sit (120 mg/kg) via oral gavage daily. CD45 (A) and MPO (B) expression was detected by indirect IF staining, and CD11b/F4/80 (C) expression was detected by direct IF double staining in lung and liver metastatic tissues. Nuclei were counterstained with DAPI. Data are presented as mean ± SD of three independent experiments. Representative images are shown. Scale bar:50 μm. *p < 0.05, **p < 0.01 and ***p < 0.001 between the indicated groups determined by the one-way analysis of variance (ANOVA). [file Image_4.tif]

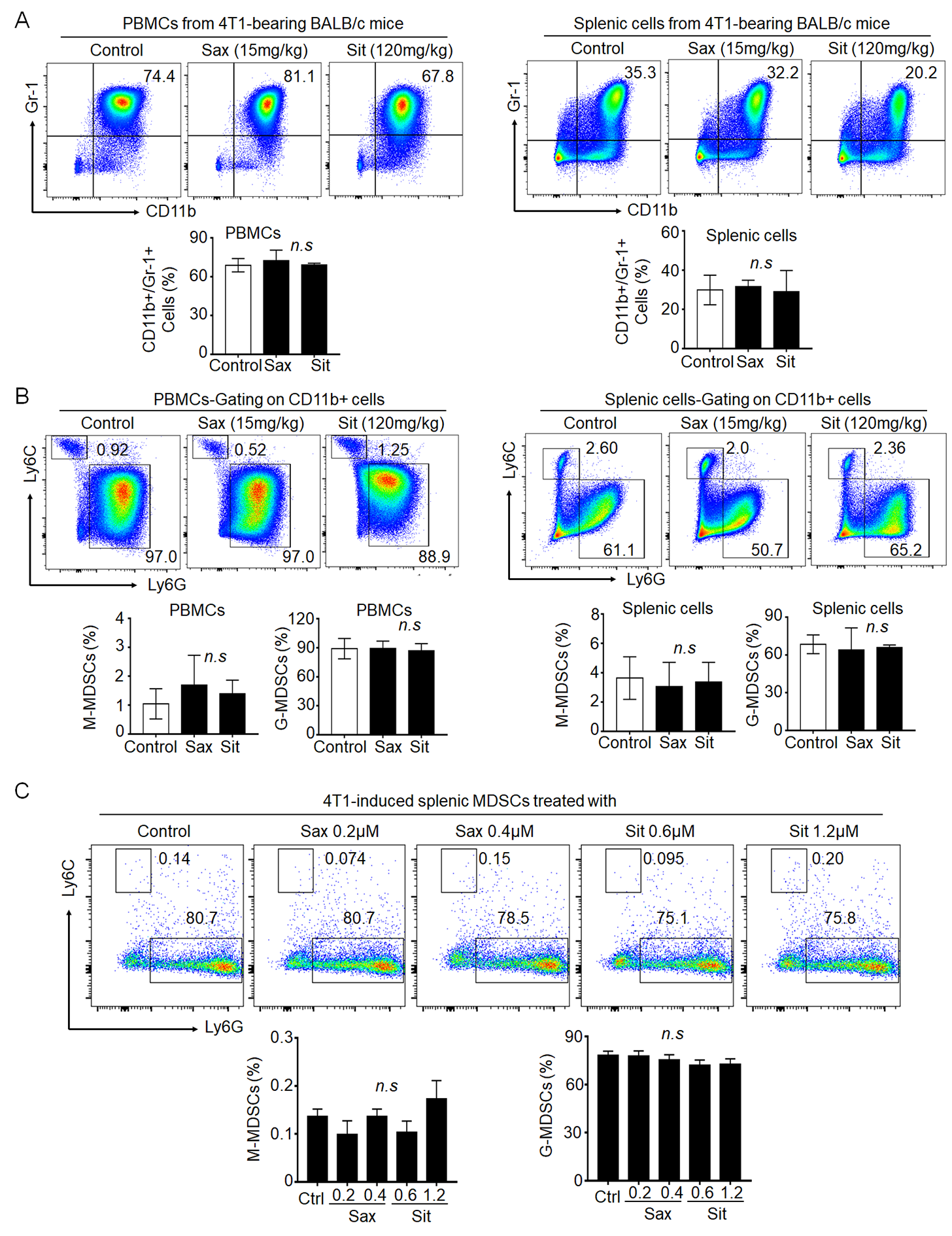

Supplement: Supplementary Figure S5 — DPP-4i does not alter MDSCs differentiation in vitro and in vivo. (A, B) DPP-4i does not alter MDSCs differentiation in vivo. 4T1-bearing BALB/c mice were treated with Sax (15 mg/kg) or Sit (120 mg/kg) via oral gavage daily. Cell percentage of CD11b+Gr-1+ MDSCs in PBMCs or Splenic cells was evaluated by flow cytometry (A). Cell percentage of Mo-MDSCs (Ly6ChiLy6G-) and G-MDSCs (Ly6CloLy6G+) in PBMCs or splenic cells was evaluated by gating on CD11b+ population using flow cytometry (B). (C) DPP-4i does not alter MDSCs differentiation in vitro. CD11b+Gr-1+ MDSCs from splenic cells of 4T1-bearing BALB/c mice were co-cultured with Sax (0, 0.2 μM, 0.4 μM) or Sit (0, 0.6 μM, 1.2 μM) in the presence of GM-CSF (10 ng/ml) for 72-96h. MDSCs differentiation was evaluated by analyzing Mo-MDSCs and G-MDSCs using flow cytometry. Data are presented as mean ± SD of three independent experiments. *p < 0.05, **p < 0.01 and ***p < 0.001 between the indicated groups determined by the one-way analysis of variance (ANOVA). [file Image_5.tif]

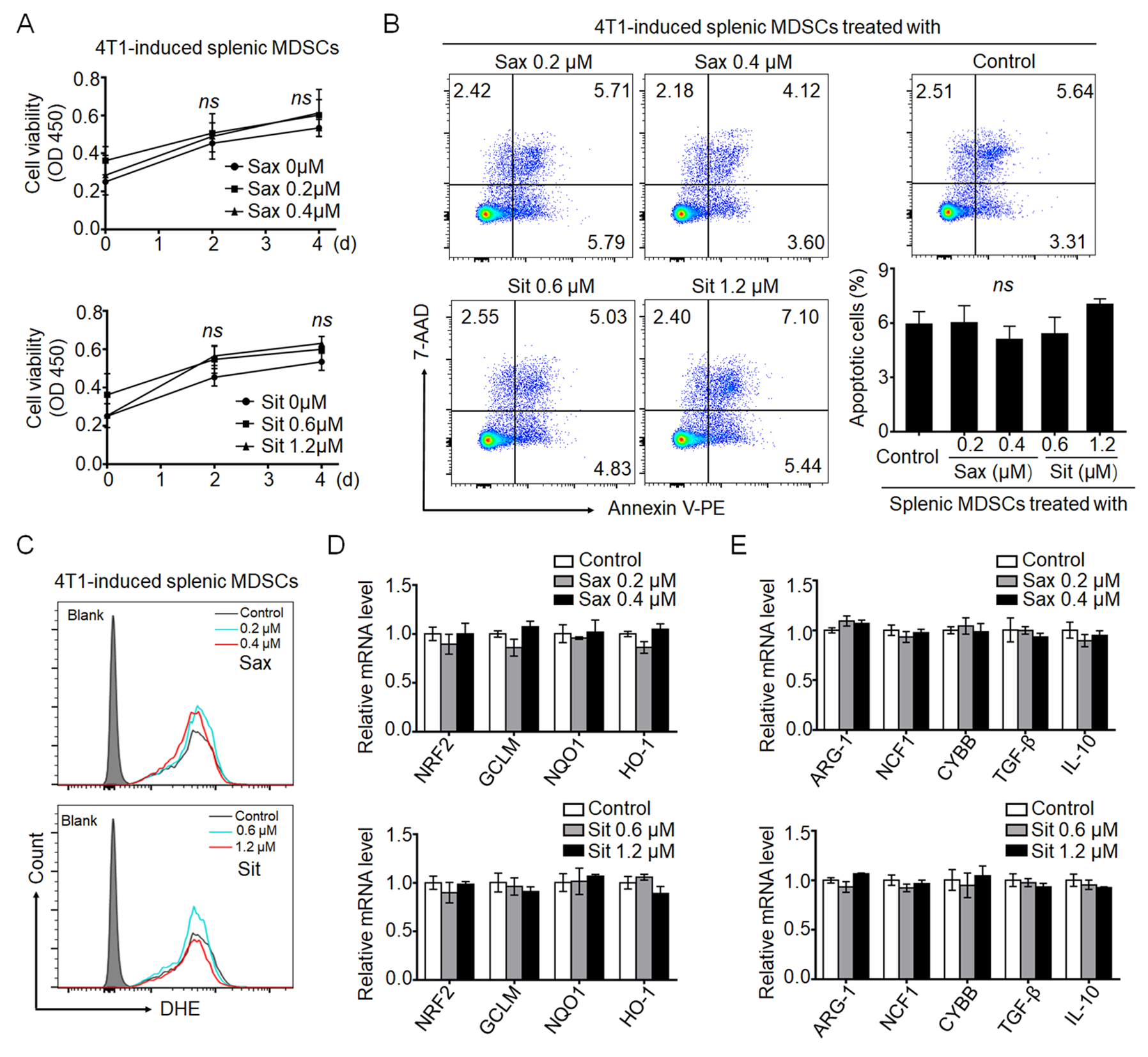

Supplement: Supplementary Figure S6 — DPP-4i treatment does not alter cell viability, apoptosis, ROS production, NRF2 activation and immune-suppressive molecules in MDSCs in vitro. CD11b+Gr-1+ MDSCs isolated from splenic cells of 4T1-bearing BALB/c mice were co-cultured with Sax (0, 0.2 μM, 0.4 μM) or Sit (0, 0.6 μM, 1.2 μM) in the presence of GM-CSF (10 ng/ml) for 24-96 h. Cell viability (co-cultured for 24-96 h) was detected by CCK-8 assay (A) and cell apoptosis (co-cultured for 72-96 h) was detected by Annexin-V-PE/7-AAD staining (B), respectively. Intracellular ROS and mROS in DPP-4i-treated MDSCs were detected using DHE and MitoSox staining by flow cytometry (C) respectively. The expressions of NRF2-responsive genes (D) and immune-suppressive genes (E) were detected by qRT-PCR. β-actin was as an internal control. Data are presented as mean ± SD of three independent experiments. *p < 0.05, **p < 0.01 and ***p < 0.001 between the indicated groups determined by the one-way analysis of variance (ANOVA). [file Image_6.tif]

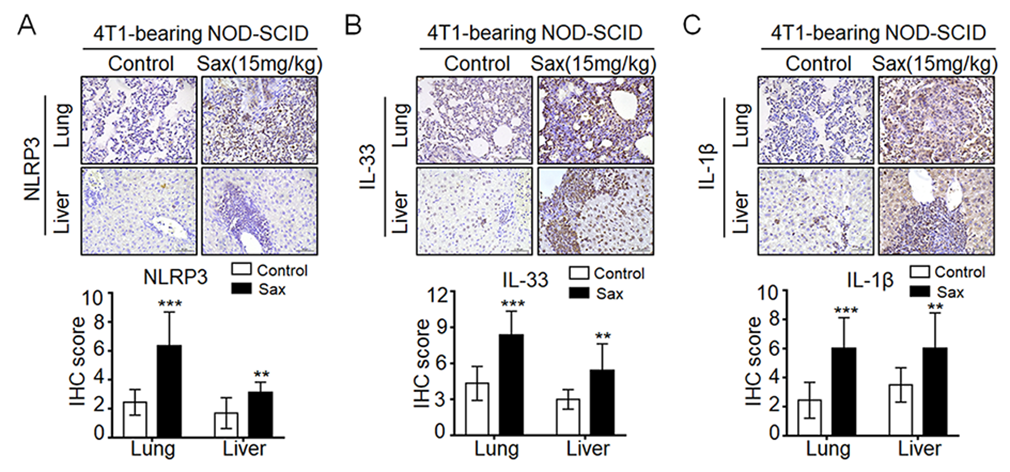

Supplement: Supplementary Figure S7 — Sax triggers NLRP3 activation of BC cells in NOD-SCID mice. 4T1-bearing NOD-SCID mice were treated with Sax (15 mg/kg) via oral gavage daily. IHC staining was performed to detect NLRP3 (A), IL-33 (B) and IL-1β (C) expression in lung and liver metastatic tissues. Data are presented as mean ± SD of three independent experiments. Representative images are shown. Scale bar: 50 μm. *p < 0.05, **p < 0.01 and ***p < 0.001 between the indicated groups determined by unpaired student’s t-test. [file Image_7.tif]

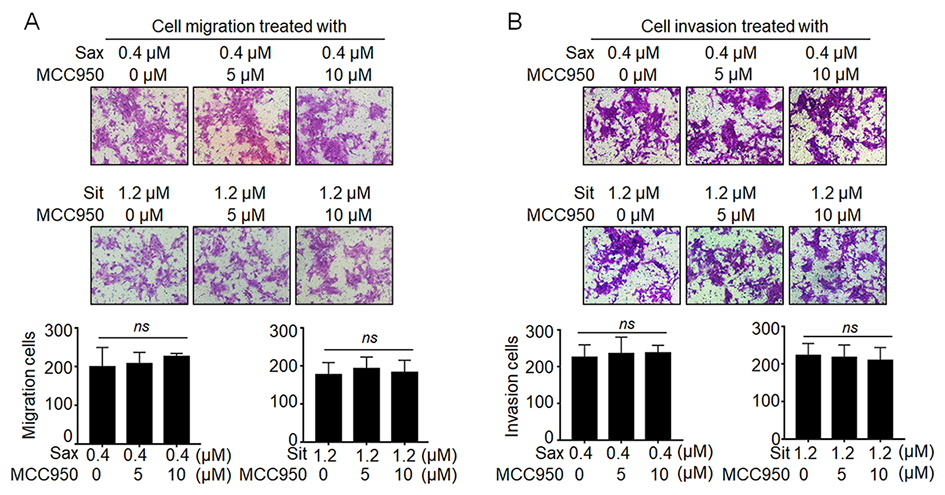

Supplement: Supplementary Figure S8 — Inhibition of NLRP3 activation do not suppress BC migration and invasion in vitro. 4T1 cells were subject to cell migration (A) and cell invasion (B) analysis upon co-treatment of Sax (0.4 μM) or Sit (1.2 μM) and NLRP3 inhibitor MCC950 (0, 5 μM,10 μM) for 24h respectively. Migration or invasion cells were counted in 5-10 random fields (200×magnification). Data are presented as mean ± SD of three independent experiments. Representative images are shown. *p < 0.05, **p < 0.01 and ***p < 0.001 between the indicated groups determined by the one-way analysis of variance (ANOVA). [file Image_8.tif]

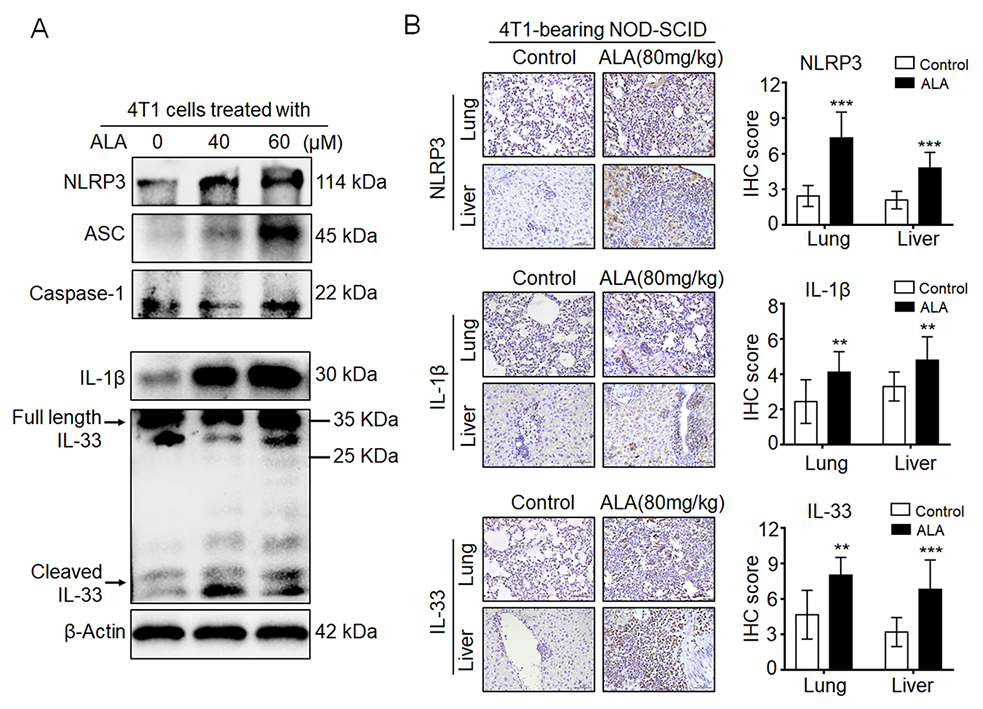

Supplement: Supplementary Figure S9 — ALA promotes NLRP3 inflammasome activation of BC cells in vitro and in vivo. (A) NRF2 activator ALA induces NLRP3 inflammasome activation in vitro. 4T1 cells were treated with ALA (0, 40 μM, 60 μM) for 4-6 h, and NLRP3 inflammasome-associated proteins were detected by western blotting. β-actin was a loading control. (B) ALA enhances NLRP3 inflammasome of BC cells in vivo. 4T1-bearing NOD-SCID mice were treated with or without ALA (80 mg/kg) via intraperitoneal (i.p.) administration. NLRP3, IL-1β and IL-33 expressions were detected by IHC staining in lung and liver metastatic tissues. Data are presented as mean ± SD of three independent experiments. Scale bar: 50 μm. *p < 0.05, **p < 0.01 and ***p < 0.001 between the indicated groups determined by unpaired student’s t-test. [file Image_9.tif]
