# Supplementary material for: Antidiabetic DPP-4 Inhibitors Reprogram Tumor Microenvironment That Facilitates Murine Breast Cancer Metastasis Through Interaction With Cancer Cells via a ROS–NF-кB–NLRP3 Axis
Source: Front Oncol. 2021 Sep 24;11:728047. doi: 10.3389/fonc.2021.728047 (PMC8497989; doi:10.3389/fonc.2021.728047)
Supplement: Supplementary file 10 [file Table_1.docx]

Supplementary Table S1 Mouse primers for qRT-PCR in this study

| Gene (GenBank  Accession No) | Primer sequences  (5'-3') | Annealing  Temp (℃) | | | Size  (bp) |
| --- | --- | --- | --- | --- | --- |
| NRF2  (NM_010902.4) | F: GCCCAGCACATCCAGACAG  R: TATCCAGGGCAAGCGACTCA | | 60 | 154 | |
| NQO1  (NM_008706.5) | F: ATGAAGGAGGCTGCTGTAGAG  R: ATATGCTAGAGATGACTCGGAAGG | | 60 | 165 | |
| GCLM  (NM_008129.4) | F: CACAATGACCCGAAAGAACTG  R: GTAGCCTTTAGACTTGATGATTCC | | 60 | 75 | |
| HO-1  (NM_010442.2)  ARG-1  (NM_007482.3)  NCF1  (NM_010876.4)  CYBB  （NM_007807.5）  TGF-β  (NM_011577.2)  IL-10  (NM_010548.2)  GM-CSF  (NM_009969.4)  G-CSF  (NM_009971.1)  M-CSF  (NM_007778.4)  β-Actin  (NM_007393) | F: CACAAAGACCAGAGTCCCTCA  R: GCAGTATCTTGCACCAGGCT  F: TACAAGACAGGGCTCCTTTCAG  R: CGCATTCACAGTCACTTAGGTG  F: ACACCTTCATTCGCCATATTGC  R: TCGGTGAATTTTCTGTAGACCAC  F: TCACATCCTCTACCAAAACC  R: CCTTTATTTTTCCCCATTCT  F: CCACCTGCAAGACCATCGAC  R: CTGGCGAGCCTTAGTTTGGAC  F: GCATGGCCCAGAAATCAAGG  R: ACACCTTGGTCTTGGAGCTTATTA  F: TGCCTGTCACGTTGAATGAAG  R: GAAATTGCCCCGTAGACCCT  F: CATGAAGCTAATGGCCCTGC  R: GGCCTGGATCTTCCTCACTT  F: AAAGGATTCTATGCTGGGCAC  R: GCTGCTTCTTTCATCCAGTCT  F: GGCTGTATTCCCCTCCATCG  R: CCAGTTGGTAACAATGCCATGT | | 60  60  60  60  60  60  60  60  60  60 | 71  191  130  198  91  162  116  160  150  154 | |

F: Forward; R: Reverse;
