# Supplementary material for: Antidiabetic DPP-4 Inhibitors Reprogram Tumor Microenvironment That Facilitates Murine Breast Cancer Metastasis Through Interaction With Cancer Cells via a ROS–NF-кB–NLRP3 Axis
Source: Front Oncol. 2021 Sep 24;11:728047. doi: 10.3389/fonc.2021.728047 (PMC8497989; doi:10.3389/fonc.2021.728047)
Supplement: Supplementary file 11 [file Table_2.doc]

Supplementary Table S2 Antibodies used for IHC and western blot in this study

| Antibodies | Catalog Number | | Dilution  for IHC | Dilution  for Wb |
| --- | --- | --- | --- | --- |
| p65 | Bioss Inc | bs-20355R | 1:500 | 1:500 |
| p-p65(ser536) | Santa Cruz | sc-101752 | 1:150 | 1:300 |
| p-IKKα/β | Cell Signaling | 16A6 | / | 1:1000 |
| IKKα | Santa Cruz | sc-52932 | / | 1:500 |
| p-IKBα | Bioss Inc | bs-2513R | / | 1:500 |
| IKBα | Bioss Inc | bs-1287R | / | 1:500 |
| TNFα | Bioss Inc | bsm-33207m | 1:400 | 1:500 |
| IL-6 | Bioss Inc | bs-4539R | 1:400 | 1:500 |
| VEGF | Bioss Inc | bs-0279R | 1:400 | 1:500 |
| MMP-2 | Bioss Inc | bs-0412R | 1:400 | 1:500 |
| MMP-9 | Bioss Inc | bs-4593R | 1:400 | 1:500 |
| ICAM-1 | Bioss Inc | bs-4618R | 1:400 | 1:500 |
| VCAM-1 | Bioss Inc | bs-0396R | 1:200 | 1:500 |
| NLRP3 | Bioss Inc | bs-10021R | 1:400 | 1:500 |
| ASC | Bioss Inc | bs-6741R | / | 1:500 |
| Caspase-1(D-3) | Santa Cruz | sc-392736 | / | 1:300 |
| IL-1β | Bioss Inc | bs-0812R | 1:400 | 1:500 |
| IL-33 | Bioss Inc | bs-2633R | 1:400 | 1:500 |
| GM-CSF | Bioss Inc | bs-3790R | 1:500 | 1:500 |
| Foxp3 | Biolegend | 623801 | 1:100 | / |
| NRF2 | Bioss Inc | bs-1074R | 1:500 | / |
| p-NRF2 | Bioss Inc | bs-2013R | 1:500 | / |
| HO-1 | Santa Cruz | sc-390991 | 1:300 | / |
| Vimentin | Bioss Inc | bs-0756R | 1:400 | / |
| β-Actin | Sigma | A5441 | / | 1:7000 |
