# Supplementary material for: Antidiabetic DPP-4 Inhibitors Reprogram Tumor Microenvironment That Facilitates Murine Breast Cancer Metastasis Through Interaction With Cancer Cells via a ROS–NF-кB–NLRP3 Axis
Source: Front Oncol. 2021 Sep 24;11:728047. doi: 10.3389/fonc.2021.728047 (PMC8497989; doi:10.3389/fonc.2021.728047)
Supplement: Supplementary file 12 [file Table_3.doc]

Supplementary Table S3 Antibodies used for FACS and IF in this study

| Antibodies | Catalog Number | | Dilution  for FACS | Dilution  for IF |
| --- | --- | --- | --- | --- |
| MPO | Boster Biotech | PB0072 | / | 1:200 |
| CD45 | Biolegend | 103101 | / | 1:100 |
| CD4 | Biolegend | 100401 | / | 1:100 |
| CD8 | Biolegend | 100701 | / | 1:100 |
| CD11b | Biolegend | 101201 | / | 1:100 |
| CD11b-FITC | Biolegend | 101206 | 1:100 | 1:50 |
| F4/80-APC | Biolegend | 123116 | / | 1:50 |
| GR-1-APC | Biolegend | 108412 | 1:100 | 1:50 |
| GR-1-APC-Cy7 | Biolegend | 108424 | 1:100 | / |
| Ly6G-PE | Biolegend | 127607 | 1:100 | / |
| Ly6G-PerCP/Cy5.5 | Biolegend | 127616 | 1:100 | / |
| Ly6C-BV421 | Biolegend | 128031 | 1:100 | / |
| Ly6C-PE/Cy7 | Biolegend | 128018 | 1:100 | / |
